# Differential cytokine signature profiles in neonates, infants, and children with enterovirus meningitis

**DOI:** 10.1128/jvi.01871-25

**Published:** 2026-05-11

**Authors:** Hélène Chabrolles, Léa Gaume, Bruno Pereira, Jérémy Lafolie, Pascale Gueirard, Anne Sophie L’Honneur, Marie Noelle Adam, Sylvie Nathanson, Stéphanie Marque-Juillet, Fouad Madhi, Matthieu Verdan, Marie Aline Guitteny, Gisele Lagathu, Jean-Luc Bailly, Cécile Henquell, Christine Archimbaud, on behalf of the Blood Enterovirus Diagnosis Infection (BLEDI) Group in the Pediatric Population Study Team

**Affiliations:** 1 Laboratory Microorganisms: Genome and Environment (LMGE), Clermont Auvergne University, CNRS UMR 6023 27006, Clermont-Ferrand, France; 2 Virology Department, National Reference Centre for Enterovirus and Parechovirus, University Hospital of Clermont-Ferrand 720120, Clermont-Ferrand, France; 3 Clinical Research and Innovation Direction - Biostatistics, University Hospital of Clermont Ferrand 55174 , Clermont-Ferrand, France; 4 Laboratoire de Biologie, Centre Hospitalier Vichy 54992 , Vichy, France; 5 Service de Virologie, Assistance Publique–Hôpitaux de Paris (AP-HP), Hôpital Cochin 518837 , Paris, France; 6 Laboratoire de Microbiologie, Centre Hospitalier Sud Francilien 55461, Corbeil-Essonnes, France; 7 Service de Pédiatrie, Centre Hospitalier de Versailles André Mignot 26938, Le Chesnay, France; 8 Service Biologie, Unité de Microbiologie, Centre Hospitalier de Versailles André Mignot 26938, Le Chesnay, France; 9 Service de Pédiatrie Générale, Centre Hospitalier Intercommunal Créteil 26949, Créteil, France; 10 Service de Pédiatrie, University Hospital of Clermont-Ferrand 55174 , Clermont-Ferrand, France; 11 Service de Pédiatrie, Hôpital Sud, CHU de Rennes 36684, Rennes, France; 12 Laboratoire de Virologie, Hôpital Sud, CHU de Rennes 679507 , Rennes, France; The University of Texas Southwestern Medical Center, Dallas, Texas, USA

**Keywords:** enterovirus meningitis, cytokine-chemokine, inflammatory response, plasma, CSF, pediatric

## Abstract

Enteroviruses (EVs) are the most frequent cause of pediatric aseptic meningitis, and cause neurological complications, such as encephalitis and flaccid paralysis. Our aim was to characterize the age-specific cytokine and chemokine response profiles in the cerebrospinal fluid (CSF) and blood of neonates, infants, and children with enterovirus meningitis. We performed an ancillary study to the French multicenter BLEDI study. We selected 163 patients with confirmed EV or suspected meningitis. Demographic, clinical, and laboratory data were analyzed. Twenty-seven cytokine/chemokines were simultaneously quantified in the CSF and plasma of the patients, using a bio-plex multiplex immunoassay. Cytokine-chemokine expression increased in the CSF with the age of EV patients compared with controls: CSF IP-10, IL-6, and IL-1ra increased 13-, 11-, 5-fold, respectively, in neonates, 41-, 53-, and 20-fold in infants, and 52-, 168-, and 69-fold in children. In plasma, cytokine/chemokines were overexpressed in neonates and downregulated in children, compared with their respective controls: plasma MCP-1, IP-10, and IL-1ra increased 10-fold in neonates, and decreased threefold in children. The expression of cytokine/chemokines varied with CSF pleocytosis but not with EV types and viral load. These findings show that cytokine and chemokine responses during EV meningitis are strongly age-dependent and should be interpreted according to patient age, with neonates, infants, and children being considered separately. Our results also indicate that age stratification is essential in future studies aiming to evaluate cytokines and chemokines as biomarkers of disease severity in EV-associated neurological infections.

**IMPORTANCE:**

Although enteroviruses (EVs) are the main cause of pediatric viral meningitis, the age-specific immune response to infection remains poorly understood. Our work is novel in combining the study of paired biological compartments (cerebrospinal fluid [CSF] and plasma), age stratification, pleocytosis status, viral load, and EV genotype, which allows a comprehensive assessment of how age shapes both local and systemic immune responses during EV meningitis. This study shows that the immunological response to EV meningitis in CSF and plasma evolves with the age of the patients. The expression of cytokines/chemokines varied with CSF pleocytosis but not with EV types and viral load. Our findings highlight that neonatal EV meningitis is associated with distinct immunopathogenic features that reflect age-dependent immune responses. These results indicate that age stratification is essential in future studies aiming to evaluate cytokines and chemokines as biomarkers of disease severity in EV-associated neurological infections.

## INTRODUCTION

Enteroviruses (EVs) are the most frequent cause of acute aseptic meningitis in the pediatric population and are attributed to more than 75% of viral meningitis cases (1, 2). Detection of EV by RT-PCR from the cerebrospinal fluid (CSF) is the gold standard for diagnosis of meningitis caused by EV, and we have shown that the detection of EV in the blood enhances diagnostic yield in children under the age of 2 years (3). No specific approved treatment is available for EV infections, and clinicians lack biomarkers of disease severity in children with enterovirus-related neurological manifestations, which range from meningitis to fatal encephalitis. Cytokine/chemokines are promising biomarkers for the prognosis of viral infections. During central nervous system (CNS) infection, host immune response plays a major role as the first line of defense and also in outcome and disease severity (4, 5). However, its molecular and cellular function is weak in neonates who have immature leukocytes and CNS immune resident cells (such as microglia, astrocytes), which results in impaired signaling pathways and reduced response compared with older children and adults (6–9). Cytokine-chemokine expression during EV meningitis was explored in patients aged 16 years or younger, without systematically distinguishing between age groups, such as neonates, infants, and children (10–13). These studies established that EV infection of the central nervous system is associated with increased levels of pro-inflammatory cytokine/chemokines, such as IL-6, IL-8, TNF-α, IL-1β, IFN-γ, IP-10, and MCP-1. Clinical and virological studies show that EV meningitis predominantly affects infants and young children, with the most severe forms (encephalitis and neonatal systemic involvement) occurring mainly in neonates. Previous studies also compared cytokine-chemokine expression in EV meningitis patients with or without pleocytosis, regardless of age (12, 13). Only one study explored the effect of age and showed a similar profile of cytokine-chemokine expression in patients under and over 2 months old with EV-related encephalitis (14). The cytokine-chemokine response to EV has been mainly studied in EV-A71, which induces a “cytokine storm” that is associated with severe neurological outcomes such as rhombencephalitis (15). Most studies dealt with the detection of cytokine/chemokines in mild and severe EV-A71 infections in pediatric patients. In most publications, cytokine-chemokine production was investigated in the CSF of patients exhibiting neurological manifestations or in the blood from hand-foot-and-mouth disease (HFMD), which is associated with mild or severe EV-A71 infections in pediatric patients (16–20). Two studies analyzed the cytokine-chemokine response in both the CSF and serum of children (median age 2 to 3 years) (21, 22). Comparison of these studies should be made with caution because the characteristics of infected patients (clinical injuries, age, presence or not of a CSF pleocytosis) vary, as do the techniques for cytokine-chemokine detection (multiplex immunoassay vs. ELISA), detection threshold, and the characteristics of the control groups tested. In addition, cytokine-chemokine responses are rarely analyzed simultaneously in paired CSF and plasma samples.

Consequently, how age influences local and systemic cytokine-chemokine responses during EV meningitis remains poorly defined. To address this gap, the present study aims to provide a quantitative, age-stratified analysis of cytokine-chemokine expression in paired CSF and plasma samples from neonates, infants, and children with enterovirus meningitis, while also taking into account pleocytosis, viral load, and EV genotype.

## MATERIALS AND METHODS

### Study design and patients

From the prospective, multicenter, observational study Blood Enterovirus Diagnosis Infection (BLEDI) performed between 1 June 2015 and 31 October 2015 and between 1 June 2016 and 31 October 2016 (3), 163 patients were selected: 29 neonates aged ≤28 days, 44 infants aged >28 days to ≤2 years, and 90 children aged >2 years to ≤16 years who had been admitted for fever without source, sepsis-like disease, or suspected meningitis. Enterovirus RT-PCR testing was done in plasma and CSF specimens. We enrolled 108 patients with confirmed EV meningitis (EV group) and 55 non-infected patients who were negative for EV in CSF and blood and for other infections tested in laboratory specimens (control group). We defined EV meningitis as either a positive EV RT-PCR assay in CSF, or in the plasma with the presence of age-specific CSF pleocytosis or at least two of the following neurological signs or symptoms: headache, nuchal rigidity, photophobia, bulging fontanelle, irritability, lethargy, vomiting, or positive Kerning’s or Brudzinsky’s sign. CSF pleocytosis was defined as a white-blood-cell (WBC) count in the CSF of more than 19/µL for neonates (aged ≤28 days), 10/µL or more for infants (aged 29–56 days), and 5/µL or more for older children (aged >56 days) (23).

In both the EV and control groups, exclusion criteria were absence or insufficient volume of plasma/CSF for cytokine analysis, and bacterial or other viral pathogens detected in biological specimens (blood, CSF, urine, respiratory samples, or stools). Patients were excluded from the EV group when viral load and genotyping results were not available, and from the control group when they had CSF pleocytosis or an elevated C-reactive protein (CRP) level in the blood (>15 mg/L).

### Procedures

Within 24 h of admission, a physician completed a standardized questionnaire for every patient, recording the nature and duration of preadmission symptoms and signs, and the results of the physical examination performed at the time of admission. Laboratory findings included the date and time of biological specimen sampling, CSF and blood count characteristics, CRP assay result, and the results of other bacteriological or virological testing of samples recorded by biologists. Details of the biological data have been previously reported (3). EV molecular diagnosis in blood and CSF specimens was performed with commercial or “in-house” RT-PCR assays (3). Viral loads were measured from EV-positive specimens, and EV strains were genotyped by the laboratory of the National Reference Center for Enteroviruses and Parechoviruses in Clermont-Ferrand (France), as previously reported (24, 25).

### Multiplex cytokine-chemokine assay

Twenty-seven cytokines and chemokines (IL-1β, -1ra, -2, -4, -5, -6, -7, -8, -9, -10, -12, -13, -15, -17, MCP-1, MIP-1a, MIP-1b, eotaxin, IP-10, RANTES, IFN-γ, TNF-α, G-CSF, GM-CSF, VEGF, FGF basic, and PDGF-bb; for functional classification, Table S1) were quantified in the plasma and the CSF (stored at −80°C) with the magnetic bead-based multiplex immunoassay Bio-Plex Pro Human Cytokine 27-plex assay (Bio-Rad, Hercules, CA), following the manufacturer’s instructions. Fluorescent signals were acquired on the Bio-Plex 200 system (Bio-Rad), and data were analyzed with Bio-Plex Manager Software 6.1 (Bio-Rad). Signals lower than the minimum detectable concentration were attributed the limit of detection (LOD) minus one unit for analysis; i.e., LOD [IL-1β] = 0.8 pg/mL, if sample value was <0.8, the sample was attributed 0.7 pg/mL.

### Statistical analysis

Continuous variables were compared between independent groups (such as EV positive vs control, neonates vs infants vs children, EV with pleocytosis vs EV without pleocytosis vs controls) by analysis of variance or Kruskal-Wallis test. Tukey-Kramer and Dunn’s post-hoc tests were applied. The results were expressed using fold change, effect size and ROC (receiver operating characteristic) area curve. Comparisons between groups were performed by χ or Fisher’s exact test for categorical variables. In addition, the representativeness of EV patients with EV RNA detected in the CSF (17 neonates, 22 infants, and 57 children) was analyzed with the univariate statistical tests cited above. The analyses were conducted separately for neonates, infants, and children. Correlation coefficients were used to study the relationships between quantitative variables. For comparisons of EV meningitis and control patients, a multivariate analysis adjusted for sample collection time was performed. For comparisons of EV types, multivariate analyses were performed to take into account possible confounders, adjusted for age, sample time, viral load, and pleocytosis for CSF or WBC for plasma. Finally, for paired comparisons, the Wilcoxon test was used. Statistical analyses concerning the comparisons of cytokine/chemokines were performed for every age group (neonates, infants, and children). Analyses were performed with Stata software. Two-sided tests with α = 0.05 were used. Sidak’s correction was applied to take into account multiple comparisons. The results were also expressed using effect sizes (ESs) and 95% confidence intervals and were interpreted according to the rule of thumb.

## RESULTS

### Patient characteristics and laboratory variables

We included 163 patients in the study, 108 with EV meningitis (neonates, *n* = 19; infants, *n* = 29; children, *n* = 60) and 55 controls (neonates, *n* = 10; infants, *n* = 15; children, *n* = 30). Infants and children with EV meningitis were younger than control patients (*P* = 0.028 and *P* = 0.008, respectively, Table 1). The time between onset of symptoms and lumbar or venipuncture did not differ between EV and control patients in all three age groups. However, among patients with EV meningitis, it increased significantly in children (median of 21 h for CSF and 23 h for plasma) compared with neonates and infants (7 h to 14 h for CSF and plasma). The frequency of CSF pleocytosis increased with age in EV patients, being observed in 32% of neonates, 48% of infants, and 92% of children. Blood counts of lymphocytes and monocytes were significantly lower in EV neonates (2.4 lymphocytes × 10^9^/L and 0.7 monocytes × 10^9^/L) than in control neonates (3.6 lymphocytes × 10^9^/L, *P* = 0.007 and 1.2 monocytes × 10^9^/L, *P* = 0.006). In contrast, EV children had higher neutrophil counts than controls (8.9 vs 6.3 neutrophils × 10^9^/L, *P* = 0.005).

**TABLE 1:** Characteristics of patients (neonates, infants, and children) with and without enterovirus meningitis^*a*^

Characteristic	Neonates			Infants			Children			EV-positive Global *P*-value
	EV meningitis (*n* = 19)	Control (*n* = 10)	*P*- value	EV meningitis (*n* = 29)	Control (*n* = 15)	*P*- value	EV meningitis (*n* = 60)	Control (*n* = 30)	*P*- value	
Demographics										
Age	18 days (11–24)^*b*^	13 days (12–28)	0.614	45 days (39–57)^*c*^	71 days (53–96)	0.028	6 years (5–8)^*d*^	8 years (6–11)	0.008	<0.001
Male sex	10 (53%)	4 (40%)	0.518	18 (62%)	5 (33%)	0.070	46 (77%)	19 (63%)	0.183	0.100
Sampling time										
Time between onset of symptoms and lumbar puncture (hours)	7 (3–22)	17 (8–22)	0.099	14 (8–22)^*c*^	16 (6–30)	0.520	21 (14–45)^*d*^	17 (7–47)	0.162	<0.001
Time between onset of symptoms and venipuncture (hours)	13 (6–20)	20 (14–29)	0.057	14 (7–26)^*c*^	11 (6–28)	0.931	23 (13–46)^d^	16 (5–44)	0.107	0.001
CSF profile										
Pleocytosis *n*/ (%)	6/19 (32%)	0	na	14/29 (48%)^*c*^	0	na	55/60 (92%)^*d*^	0	na	<0.001
Proteins (g/L)	0.6 (0.5–0.8)	0.5 (0.5–0.6)	0.155	0.5 (0.4–0.8)^*c*^	0.3 (0.2–0.4)	0.003	0.3 (0.2–0.4)^*d*^	0.2 (0.2–0.2)	0.0001	<0.001
Glucose (mmol/L)	2.7 (2.5–3.1)	2.8 (2.7–2.9)	0.566	3 (2.7–3.4)^*c*^	3.1 (2.8–3.3)	0.823	3.6 (3.3–4.0)^*d*^	3.6 (3.3–3.9)	0.878	<0.001
Blood profile										
WBC (x 10^9^/L)	8.7 (5.7–10.5)	9.4 (8.3–10.7)	0.291	8.1 (6.2–11.7)^*c*^	10.2 (7.3–12.4)	0.276	10.7 (8.4–13.6)^*d*^	8.8 (6.1–11.2)	0.015	0.011
Neutrophils (x 10^9^/L)	4.3 (2.6–7.3)	3.2 (2.1–3.7)	0.150	2.8 (2.0–4.6)^*c*^	3.1 (1.4–6.6)	0.595	8.9 (6.8–11.5)^*d*^	6.3 (4.2–9.0)	0.005	<0.001
Lymphocytes (x 10^9^/L)	2.4 (1.9–3.3)	3.6 (3.3–6.0)	0.007	3.6 (2.0–5.3)^*c*^	5.2 (3.8–6.4)	0.058	1.1 (0.9–1.6)^*d*^	1.0 (0.8–1.9)	0.591	<0.001
Monocytes (x 10^9^/L)	0.7 (0.5–1.0)	1.2 (0.9–1.9)	0.006	0.8 (0.6–1.3)^*c*^	0.8 (0.6–1.2)	0.970	0.6 (0.5–0.8)	0.8 (0.6–1.0)	0.058	0.039
EV characteristics										
CSF viral load (log10 copies/ml)	4.7 (4.0–5.8)	na	na	4.60 (3.8–5.4)	na	na	5.0 (4.3–5.7)	na	na	0.335
Plasma viral load (log10 copies/ml)	5.9 (4.2–7.5)	na	na	5.3 (4.5*–*7.2)^*c*^	na	na	3.8 (3.3–4.2)^d^	na	na	<0.001

^*a*^ Data are *n* (%) or median (IQR), unless otherwise indicated. Demographic and laboratory data of patients with and without EV meningitis were analyzed separately according to age category. EV, enterovirus; Control, patient not infected with enterovirus; CSF, cerebrospinal fluid; WBC, white blood cells; na, not applicable.

^*b*^ Significant difference between EV neonates and infants.

^*c*^ Significant difference between EV infants and children.

^*d*^ Significant difference between EV children and neonates.

### Differential cytokine-chemokine profiles

**Fig 1:**
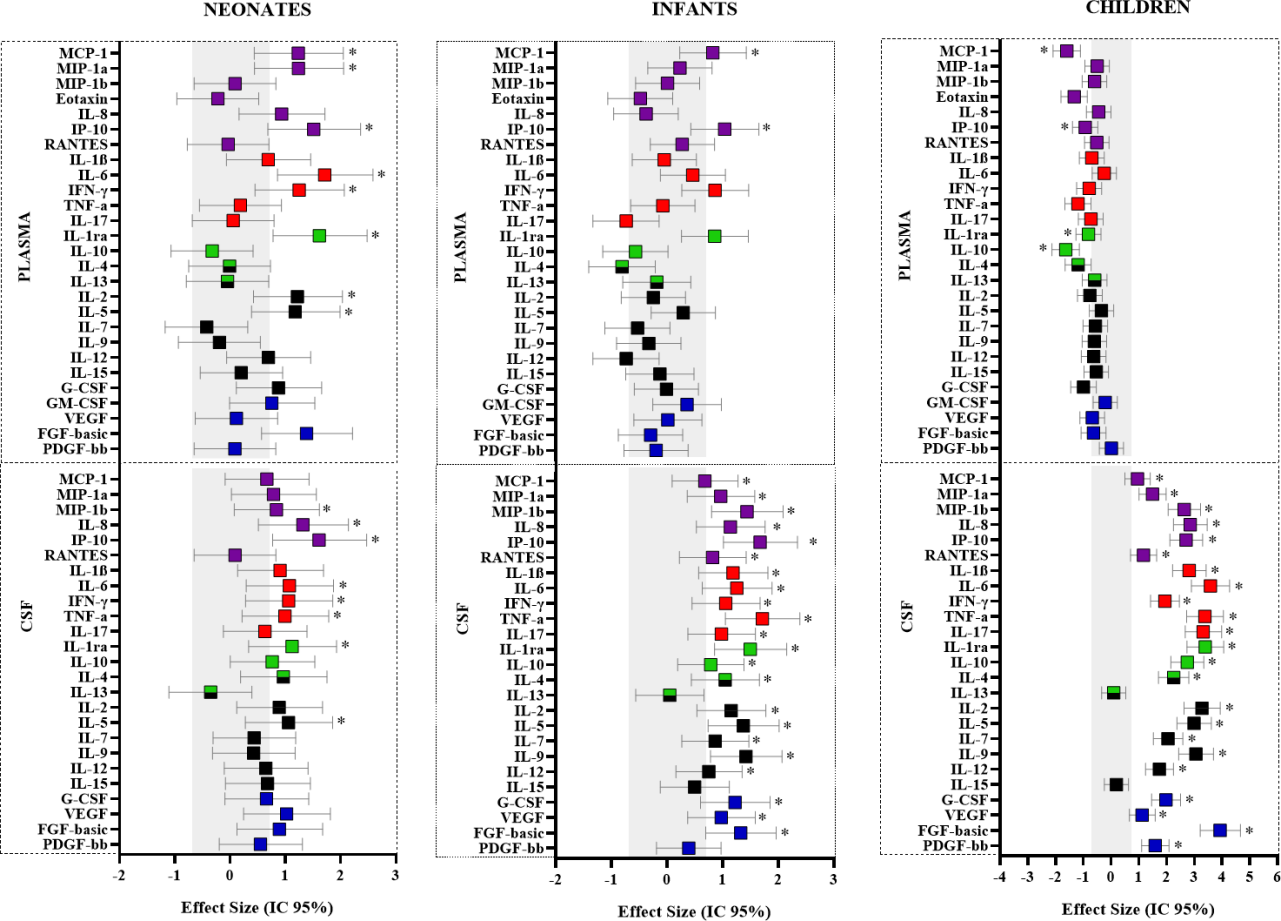
Effect size of cytokine-chemokine expression between EV and control groups in neonates, infants, and children. The gray area corresponds to effect size values between −0.8 and 0.8. Stars indicate robust differences between control and EV patients with fold change ≥ 2, effect size > |0.8|, ROC > 0.7, and *P*-value < 0.05. Eotaxin and GM-CSF were not expressed in the CSF. Purple boxes correspond to chemokines, red boxes to pro-inflammatory cytokines, green boxes to anti-inflammatory cytokines, black boxes to cytokines involved in the adaptive immunity and lymphocyte activation, and blue boxes to growth factors.

The 27 cytokine/chemokines quantified in the CSF and plasma of EV patients were differentially expressed according to age group and biological compartment. In the control patients, the levels of cytokine/chemokines were comparable in the three age groups in both CSF and plasma. In contrast, the levels of 20/27 of the cytokine/chemokines in plasma and 16/27 in CSF varied significantly by age group with EV meningitis (Table S2). Cytokine-chemokine levels in CSF were upregulated in the three age groups of EV patients compared with control patients, with an ascending increase in fold change and effect size according to age (Fig. 1; Table S3). Plasma cytokine/chemokines were mostly upregulated in EV neonates and downregulated in children, with infants having an intermediate profile (Fig. 1; Table S3). After adjustment for sample time, most differences in cytokine-chemokine expression between EV meningitis and control patients remained significant across age groups (Table S3).

We used four criteria to achieve a robust assessment of cytokine-chemokine changes between EV and control patients: a fold change (FC) in cytokine-chemokine concentration ≥2, an effect size >|0.8|, a ROC > 0.7, and a *P*-value < 0.05 (Fig. 1; Table S3). Overall, we found 12 cytokine/chemokines with significant changes between EV and control patients (Fig. S1). MIP-1b, IL-8, IP-10, IL-6, IFN-γ, TNF-α, IL-1ra, and IL-5 were statistically upregulated in the CSF of all EV patients. For example, IP-10, IL-6, IL-1ra, and IL-8 were over-expressed 13-, 11-, 5-, and 3-fold, respectively, in neonates, 41-, 53-, 20-, and 8-fold in infants, and 52-, 168-, 69-, and 26-fold in children compared with their respective controls. Plasma levels of MIP-1a, IL-6, IFN-γ, IL-2, and IL-5 were upregulated only in EV neonates. MCP-1 and IP-10 were upregulated in the plasma of EV neonates (10-fold increase, *P* < 0.009) and infants (4-fold increase, *P* < 0.014) and downregulated in children (3-fold decrease, *P* ≤ 0.001) compared with controls. Additionally, IL-1ra plasma levels increased 10-fold (*P* < 0.001) in EV neonates and decreased 2.6-fold (*P* < 0.001) in EV children. IP-10 levels (and those of IL-1ra except in the plasma of infants) were modulated in all EV patients and biological compartments (Fig. S1; Table S3).

To ensure the robustness of our results, we performed a sensitivity analysis excluding patients for whom EV RNA was not detected in the CSF. Accordingly, two neonates, seven infants, and three children in the “EV meningitis groups” were excluded. The cytokines/chemokines for which the fold change, effect size, ROC and *P*-value were all significant remained unchanged in the plasma and CSF of infants and children. In the CSF of neonates, seven additional cytokines were significant across all four criteria: MIP-1α and IL-1β, which were selected among the 12 cytokine/chemokines selected for our study, as well as IL-4, IL-12, G-CSF, FGF-basic, and PDGF-bb (Table S4).

### Correlation between expression of cytokines and chemokines

**Fig 2:**
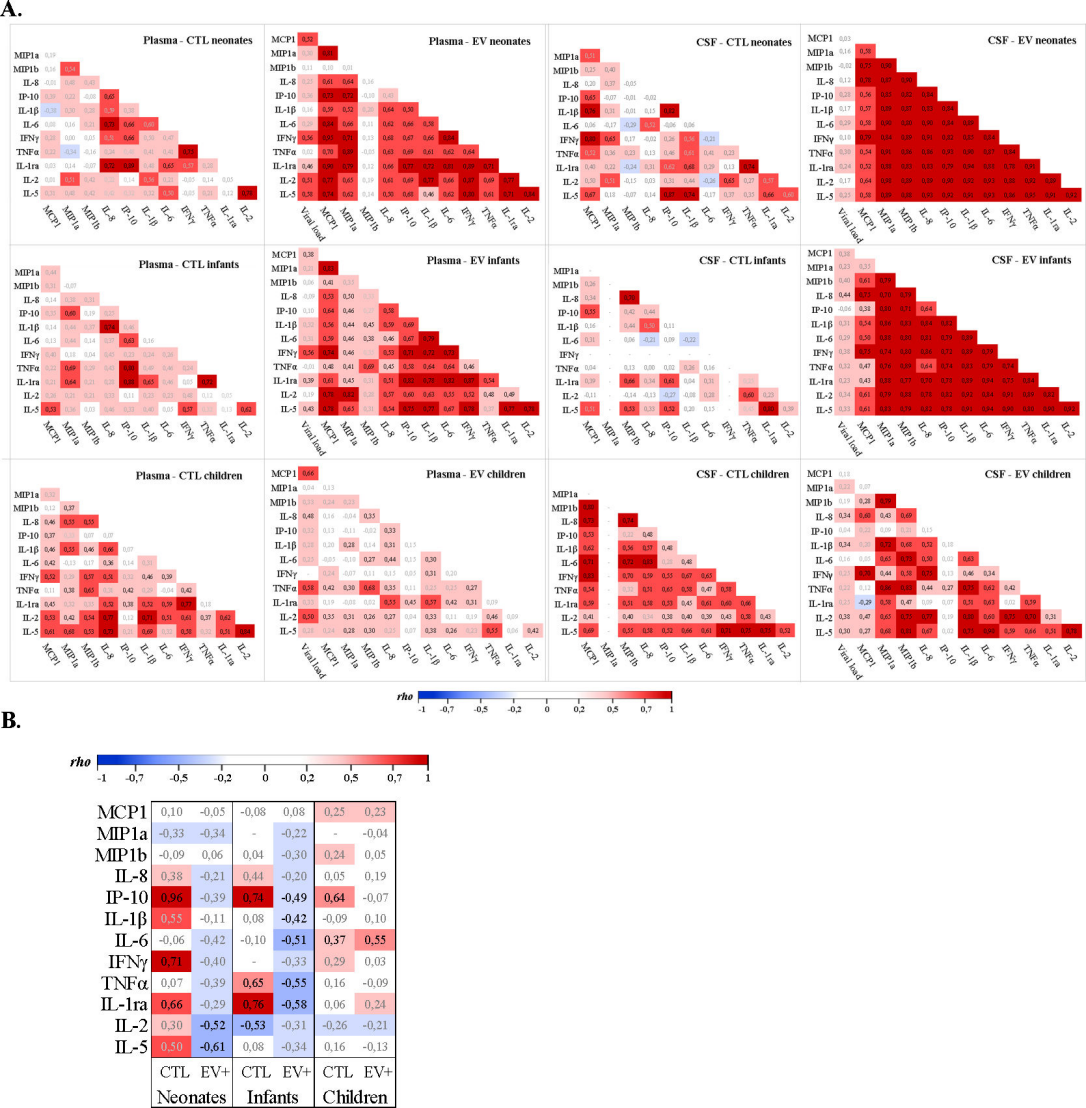
Correlation matrix of the cytokines-chemokines. The strength of the correlation between two variables is represented by the color at the intersection of those variables. Colors range from bright red (strong positive correlation; i.e., rho = 1.0) to bright blue (strong negative correlation; i.e,. rho= −1.0). Rho values are indicated at each intersection, and colored gray if *P* > 0.05 or black if *P* < 0.05. (**A**) Correlation analysis in the plasma or in the CSF of neonates, infants, and children. (**B**) Association of each cytokine-chemokine level in the plasma and the CSF. (EV: EV meningitis; CTL: control patient).

The level of each of the 12 selected cytokine/chemokines in the CSF of EV neonates and infants was positively correlated with those of other cytokine/chemokines (rho >0.7 and *P* < 0.05 for 62/66 pairwise comparisons, (Fig. 2A). In contrast, only 17 pairs of cytokine/chemokines had a strong positive association in the CSF of EV children. The levels of pro-inflammatory cytokines (IL-1β, IL-6, and IFN-γ) were positively correlated (rho > 0.7) with those of anti-inflammatory IL-1ra in the plasma of EV neonates and infants. More specifically in neonates, the levels of MCP-1 and IL-1ra had a strong positive association with each other and with MIP-1a, IP-10, IL-6, IL-2, and IL-5 plasma levels. The expression of cytokine/chemokines and viral loads in CSF were weakly associated. In the plasma, MCP-1, IFN-γ, IL-2, and IL-5 in neonates, INFγ in infants, and MCP-1, TNF-α, and IL-2 in children showed moderate association with the plasma viral load (0.5 > rho > .7) (Fig. 2A). Finally, the levels of CSF cytokine/chemokines were poorly associated with those of plasma (Fig. 2B).

### Local cytokine-chemokine profiles in the CSF

**Fig 3:**
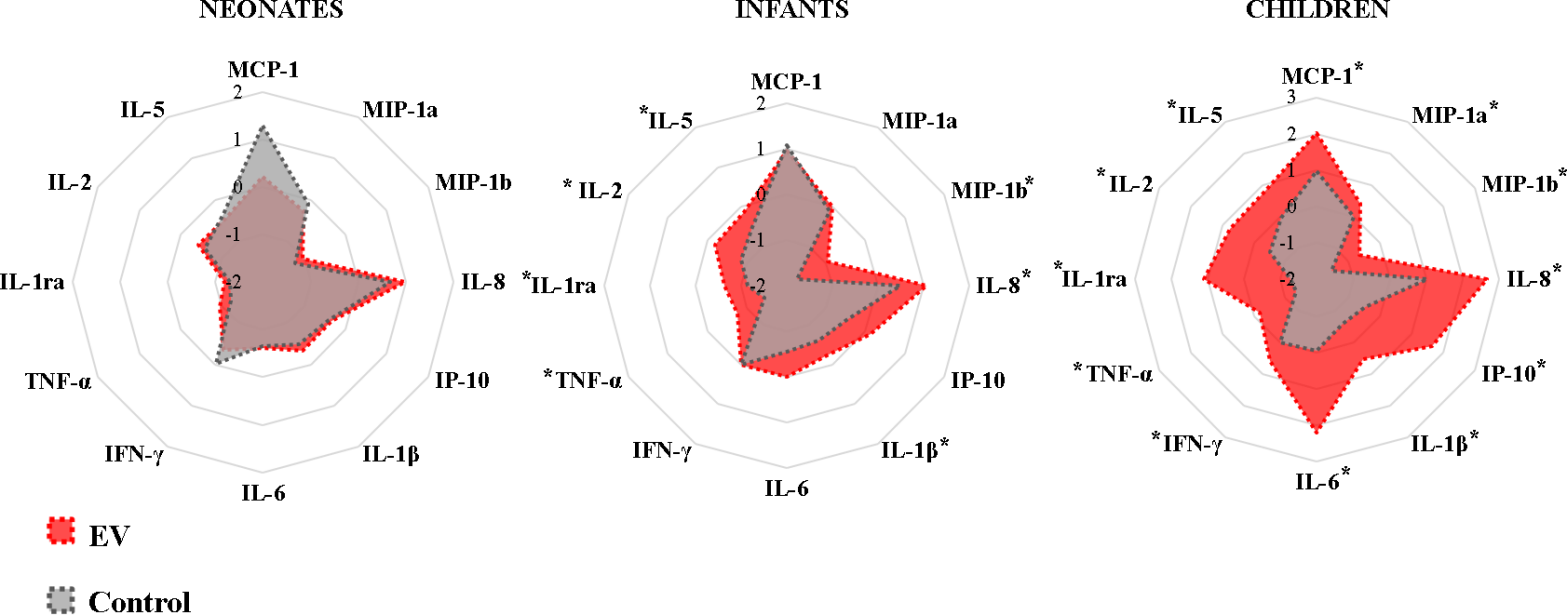
CSF-to-plasma ratios of cytokine-chemokine concentrations in neonates, infants, and children. Data are median (log_10_ pg/mL). Patients were included if the time between venipuncture and lumbar puncture was <8 h and if the red blood cell count in the CSF was <160/mm^3^. Significant difference in the ratio between EV and control patients are indicated by * (*P*-value < 0.05). EV: enterovirus meningitis; Control: patient non infected with enterovirus.

Using CSF-to-plasma cytokine-chemokine concentration ratios, we observed markedly higher CSF relative levels for several cytokines in children with EV meningitis (Fig. 3; Table 2). In EV children, IL-8, IL-6, MCP-1, and IP-10 had particularly high CSF-to-plasma ratios (457, 161, 102, and 44, respectively) compared with control patients (11, 0.9, 10, and 0.3, respectively; *P* < 0.001). In contrast, CSF-to-plasma ratios were not markedly increased in neonates and infants with EV meningitis. In infants, modest increases in CSF-to-plasma ratios were observed for MIP-1b, IL-8, IL-1β, TNF-α, IL-1ra, IL-2, and IL-5 compared with controls (*P* < 0.022), and no significant increases were detected in neonates.

**TABLE 2:** CSF/plasma ratios of cytokine-chemokine concentrations in EV and control neonates, infants, and children^*a*^

	Neonates			Infants			Children			EV positive
	EV+ (*n* = 9)	CTL (*n* = 5)	*P-*value	EV+ (*n* = 19)	CTL (*n* = 13)	*P-*value	EV+ (*n* = 51)	CTL (*n* = 27)	*P-*value	Global *P*-value
Time sympt-CSF	6 [3–14]	19 [8–19]	0.096	17 [9–25]	12 [6–24]	0.701	21 [14–49]	15 [7–36]	0.068	na
Time sympt-plasma	7 [3–12]	19 [8–24]	0.109	16 [8–26]	11 [6–23]	0.632	22 [12–50]	15 [5–36]	0.063	na
MCP-1	1.63 [1.43–38.75]	19.23 [6.87–25.75]	0.205	9.34 [3.32–24.89]^*c*^	12.33 [5.94–18.04]	0.454	102.29 [45.43–166.14]^*d*^	*9.57* [3.16–15.61]	*<*0.001	*<*0.001
MIP-1a	0.51 [0.42–5.97]	0.80 [0.58–1.00]	0.842	1.00 [0.49–3.43]^*c*^	0.77 [0.59–0.99]	0.118	2.51 [1.41–3.86]^*d*^	1.00 [0.79–1.00]	*<*0.001	*<*0.001
MIP-1b	0.09 [0.06–0.44]	0.06 [0.05–0.08]	0.317	0.11 [0.06–0.56]	0.02 [0.02–0.03]	<0.001	0.22 [0.15–0.41]^*d*^	0.03 [0.01–0.05]	<0.001	0.006
IL-8	9.51 [4.72–27.53]	4.67 [4.04–5.86]	0.125	11.47 [4.85–48.67]	3.21 [1.69–4.18]	0.001	456.55 [158.30–1088.39]	10.89 [3.08–40.46]	*<*0.001	*<*0.001
IP-10	0.45 [0.22–9.50]	0.39 [0.33–0.46]	0.842	1.35 [0.15–3.39]^*c*^	0.33 [0.17–0.48]	0.120	43.62 [28.82–61.74]^*d*^	0.34 [0.19–1.03]	*<*0.001	*<*0.001
IL-1β	0.47 [0.18–0.88]	0.32 [0.30–0.34]	0.739	0.60 [0.26–2.09]	0.25 [0.23–0.29]	0.004	3.29 [2.34–4.84]	0.26 [0.16–0.39]	*<*0.001	*<*0.001
IL-6	0.25 [0.05–246.36]	0.23 [0.12–0.31]	0.947	1.00 [0.10–66.92]	0.29 [0.10–0.45]	0.050	161.39 [72.05–265.22]	0.88 [0.24–2.08]	*<*0.001	0.025
IFN-γ	0.44 [0.23–2.44]	0.94 [0.70–1.00]	0.463	1.00 [0.64–4.98]^*c*^	1.00 [1.00–1.00]	0.622	3.89 [2.05–6.88]^*d*^	1.00 [0.93–1.02]	*<*0.001	*<*0.001
TNF-α	0.11 [0.09–0.84]	0.06 [0.05–0.08]	0.162	0.18 [0.05–0.48]^*c*^	0.04 [0.03–0.04]	*<*0.001	0.65 [0.43–0.93]^*d*^	0.05 [0.03–0.06]	*<*0.001	*<*0.001
IL-1ra	0.06 [0.05–3.60]	0.05 [0.03–0.05]	0.096	0.23 [0.04–3.12]^*c*^	0.07 [0.01–0.08]	0.022	12.33 [6.15–35.58]^*d*^	0.05 [0.02–0.17]	*<*0.001	*<*0.001
IL-2	0.36 [0.9–0.36]^*b*^	0.26 [0.17–0.79]	0.842	0.64 [0.26–1.84]^*c*^	0.14 [0.13–0.26]	0.003	5.73 [1.80–8.08]^*d*^	0.31 [0.16–0.58]	*<*0.001	*<*0.001
IL-5	0.36 [0.10–5.45]	0.46 [0.33–0.98]	0.386	0.72 [0.42–2.85]	0.30 [0.17–0.56]	0.020	6.65 [2.76–13.89]^*d*^	0.92 [0.33–1.41]	*<*0.001	*<*0.001

^*a*^ Data are median [IQR]. Patients were included if the time between the venipuncture and the lumbar puncture was <8 h and if the red blood cell count in the CSF was <160/mm^3^. EV+: EV meningitis patients; CTL: control patients, na: not applicable.

^*b*^ Significant difference in cytokine expression between EV neonates and infants.

^*c*^ Significant difference in cytokine expression between EV infants and children.

^*d*^ Significant difference in cytokine expression between EV children and neonates.

### Cytokine-chemokine profiles in the presence of CSF pleocytosis

**Fig 4:**
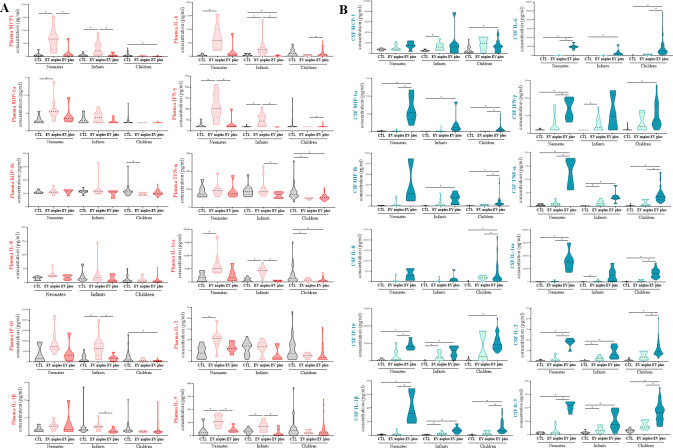
Cytokine-chemokine expression in neonates, infants, and children with EV meningitis, with or without pleocytosis, and of controls. Cytokine-chemokine expression (pg/mL) (**A**) in CSF and (**B**) in plasma. Patients were included if the time between the venipuncture and the lumbar puncture was <8 h and if the red blood cell count in the CSF was <160/mm3. EV: enterovirus, EV no pleo: EV patients without CSF pleocytosis; EV pleo: EV patients with CSF pleocytosis; CTL: control patients. *P < 0.05.

EV patients with and without pleocytosis had significant changes in the levels of cytokine/chemokines (Fig. 4). In the CSF of neonates and children, 9/12 cytokine/chemokines were upregulated in the pleocytosis group compared with the non-pleocytosis groups. For instance, IL-6 levels increased 1,300-fold in neonates and 5-fold in children, and IL-1ra increased 57-fold in neonates and 52-fold in children. A similar trend was observed in the CSF of infants. In contrast, most cytokine-chemokine concentrations were upregulated in the plasma of EV neonates and infants without pleocytosis. For instance, plasma levels of MCP-1 and IFN-γ were similar in control and EV patients with pleocytosis, but in the absence of pleocytosis, they increased in neonates and infants, 10-fold for MCP-1, 7- and 2-fold for IFN-γ, respectively. EV neonates, infants, and children with and without pleocytosis were similar in terms of age, gender, and time between onset of symptoms and lumbar puncture and venipuncture (Table S5). The peripheral lymphocyte count was significantly higher in EV neonates and infants with pleocytosis than in those without.

### Cytokine-chemokine profiles according to EV type

Of the 18 EV types identified in our patients, the six most frequent were coxsackievirus B5 (CB5; *n* = 18), echovirus 6 (E6; *n* = 17), E9 (*n* = 15), E30 (*n* = 13), CA9 (*n* = 9), and E5 (*n* = 8) (Table 3). Patients infected with these EVs were similar in terms of age and time of CSF/plasma sampling. WBC count in the CSF was significantly higher in patients infected with E6, E30, CB5, and CA9. The EV types were associated with different cytokine-chemokine profiles. The CSF levels of IL-8, IP-10, TNF-α, IL-1β, IL-6, IL-1ra, and IL-5 were different according to the EV types. In plasma, five cytokine/chemokines (MCP-1, IP-10, TNF-α, IL-2, and IL−5) were differentially expressed according to the EV types. The variations in cytokine-chemokine levels according to the EV types were associated with pleocytosis in CSF and patient age in plasma (multivariate analysis, Table 3).

**TABLE 3:** Cytokine-chemokine expression by EV types^*a*^

Characteristic	E6	E30	CB5	E5	E9	CA9	*P*-value	Multivariate analysis adjusted for:			
								Age	Sample time	Viral load	WBC
Demographics											
Patients (*n*)	17	13	18	8	15	9	na				
Age	5.7 years [5.1y–7.3y]	5.9 years [5.7y–8.5y]	3.1 years [1.2m–5.9y]	2.6 years [38d–6.4y]	3.7 years [1.5m–6.2y]	4.5 years [37d–6.0y]	0.055				
Neonates (*n*)	1	1	3	1	1	1	na				
Infants (*n*)	0	0	5	3	5	3	na				
Children (*n*)	16	12	10	4	9	5	na				
Time between onset of symptoms and lumbar puncture (hours)	17 [14–28]	19 [14–23]	18 [9–64]	21 [11–45]	13 [9–27]	17 [14–48]	0.967				
Time between onset of symptoms and venipuncture (hours)	20 [11–29]	19 [13–25]	16 [8–64]	21 [10–46]	22 [9–62]	26 [14–51]	0.979				
CSF characteristics											
Pleocytosis (n/)	17/17 (100%)	13/13 (100%)	17/18 (94%)	4/8 (50%)	10/15 (66%)	6/9 (66%)	na				
WBC (x 10^9^/L)	137 [83–224]	183 [46–410]	225 [95–920]	7 [1–49]	10 [5–37]	190 [4–510]	*<*0.001				
Viral load in CSF (log copy/mL)	5.0 [4.8–5.6]	4.9 [4.3–5.3]	4.5 [3.8–5.7]	5.2 [4.3–6.2]	5.7 [5.2–6.3]	5.1 [3.4–5.6]	0.153				
Blood characteristics											
WBC (x 10^9^/L)	11.8 [8.7–12.5]	11.6 [9.2–16.0]	10.3 [8.6–13.2]	8.4 [6.1–13.7]	7.5 [5.9–11.1]	12.8 [6.5–16.1]	0.076				
Viral load in blood (log copy/mL)	3.1 [2.36–3.4]	4.2 [3.8–5.1]	4.8 [4.2–5.7]	5.6 [4.1–6.5]	5.1 [4.2–7.4]	6.0 [4.8–8.4]	0.003				
Cytokine-chemokine expression in plasma											
MCP-1	10.0 [6.6–20.4]	11.2 [7.3–19.3]	12.5 [9.1–20.2]	44.8 [13.5–289.5]	22.0 [10.9–431.8]	26.3 [10.1–52.8]	0.030	< 0.001	ns	ns	0.015
MIP-1a	2.3 [2.3–2.3]	2.3 [2.3–2.3]	2.3 [2.3–2.6]	2.3 [2.6–4.6]	2.3 [2.3–4.0]	2.3 [2.3–2.3]	0.137	na	na	na	na
MIP-1b	194.5 [183.75–218.84]	209.5 [198.6–221.6]	213.1 [192.5–227.4]	205.4 [188.8–253.5]	225.6 [199.3–239.1]	204.3 [194.3–217.8]	0.265	na	na	na	na
IL-8	5.2 [3.4–7.0]	6.8 [3.3–12.0]	3.9 [2.0–13.1]	10.6 [6.2–23.1]	9.8 [4.8–15.1]	9.0 [5.3–18.2]	0.079	na	na	na	na
IP-10	528.6 [399.6–596.0]	396.3 [301.0–622.7]	425.5 [238.1–1259.8]	2052.1 [547.8–7289.7]	974.1 [572.0–6028.2]	587.0 [269.9–2158.4]	0.024	< 0.001	ns	ns	ns
IL-1β	2.5 [2.2–2.9]	2.1 [1.6–3.1]	2.3 [1.8–3.0]	2.6 [2.0–5.1]	2.6 [2.2–4.4]	2.2 [2.1–3.0]	0.747	na	na	na	na
IL-6	8.9 [4.5–19.1]	8.0 [3.6–26.3]	4.6 [2.3–8.6]	12.5 [4.7–76.5]	7.5 [3.4–26.3]	6.4 [1.2–8.2]	0.413	na	na	na	na
IFN-γ	19.2 [19.2–19.2]	19.2 [19.2–19.2]	19.2 [19.2–19.2]	20.7 [19.2–42.7]	19.2 [19.2–63.4]	19.2 [19.2–24.0]	0.087	na	na	na	na
TNF-α	46.8 [42.7–54.3]	54.5 [47.1–64.4]	57.0 [49.6–67.1]	72.7 [53.9–85.0]	60.4 [55.7–86.7]	56.0 [50.0–77.9]	0.034	0.001	ns	ns	ns
IL-1ra	617.8 [552.9–1519.6]	566.9 [242.9–943.0]	447.6 [44.1–795.9]	1,845.9 [503.5–4,907.1]	578.3 [245.0–3,866.7]	285.9 [170.0–1,596.7]	0.210	na	na	na	na
IL-2	10.0 [1.0–2.6]	2.6 [1.4–6.8]	2.2 [1.0–3.5]	6.8 [3.2–8.6]	4.0 [1.9–7.6]	2.3 [1.0–3.5]	0.033	*<* 0.001	ns	0.013	ns
IL-5	18.7 [11.8–24.4]	26.4 [7.0–39.2]	24.6 [9.5–30.9]	67.4 [38.4–126.5]	64.7 [26.5–110.1]	41.3 [11.4–45.8]	0.006	0.005	ns	ns	ns
Cytokine-chemokine expression in CSF											
MCP-1	1,534.4 [847.3–2,130.0]	1,315.4 [1,080.2–1,808.8]	1,258.4 [587.3–1,642.8]	1,668.4 [1,339.0–1,791.8]	1682.9 [865.0–2,314.3]	1380.6 [1,131.6–1,624.1]	0.623	na	na	na	na
MIP-1a	7.1 [5.2–15.9]	7.8 [6.0–8.9]	7.7 [3.7–31.5]	5.2 [4.2–9.6]	2.6 [2.3–6.1]	8.5 [2.3–17.7]	0.100	na	na	na	na
MIP-1b	75.4 [47.7–203.4]	63.6 [48.7–115.8]	101.3 [36.6–152.3]	63.7 [37.8–115.8]	35.5 [18.7–104.5]	45.4 [32.2–113.5]	0.201	na	na	na	na
IL-8	3,308.2 [1,704.2–5,718.9]	3,385.1 [1,260.4–5,171.1]	1,102.0 [706.3–1,500.3]	2,505.2 [752.2–5,813.2]	1,423.8 [426.6–5,573.5]	1,016.4 [414.3–2,588.9]	0.037	ns	0.011	ns	0.006
IP-10	22,968.8 [19,065.9–26,494.5]	20,171.2 [15,079.5–22,109.3]	15,576.0 [13,493.9–21,717.2]	11,693.7 [10,338.7–13,076.9]	13,434.5 [3,675.4–27,368.0]	15,307.6 [10,823.2–17,457.9]	0.002	ns	ns	ns	<0.001
IL-1β	10.7 [6.8–17.6]	17.3 [7.5–22.5]	7.97 [6.13–19.75]	5.2 [4.4–6.2]	6.49 [2.49–7.43]	7.3 [4.4–9.4]	0.005	ns	ns	ns	0.001
IL-6	1,966.2 [916.4–2,386.6]	1393.1 [618.3–2,335.9]	896.0 [352.4–2,402.9]	404.1 [45.3–1,047.9]	327.47 [21.68–924.86]	365.3 [314.4–1,560.1]	0.003	0.023	ns	ns	<0.001
IFN-γ	77.2 [61.2–177.3]	93.0 [58.0–170.7]	88.0 [50.2–119.3]	115.5 [88.4–151.1]	97.4 [20.7–141.6]	77.7 [53.1–132.1]	0.789	na	na	na	na
TNF-α	44.9 [35.6–73.3]	36.9 [33.6–49.7]	51.2 [23.0–85.3]	26.9 [18.0–33.4]	20.5 [18.9–31.0]	33.1 [27.2–42.3]	0.010	ns	0.029	ns	<0.001
IL-1ra	8,078.8 [4,730.3–9,706.7]	6,719.0 [4,053.4–8,845.0]	8,261.07 [3,240.97–12,603.30]	3,382.7 [1,050.4–7,465.1]	3,020.8 [1,055.1–4,905.2]	4,185.8 [2,018.9–9,110.9]	0.009	ns	ns	ns	<0.001
IL-2	9.8 [8.3–17.3]	10.7 [7.8–15.1]	9.29 [6.44–14.28]	7.6 [5.0–12.7]	7.00 [2.92–12.24]	8.5 [4.9–11.3]	0.097	na	na	na	na
IL-5	194.9 [133.6–231.6]	177.5 [102.4–206.2]	139.3 [104.1–240.8]	95.6 [65.9–159.3]	78.99 [65.85–168.31]	112.0 [76.6–184.4]	0.018	ns	ns	ns	<0.001

^*a*^ Data are median [IQR], unless otherwise indicated. Eighteen different EV types were identified by genotyping, three within the EV-A species (CA2, CA10 and EV-A71: five patients) and 15 within the EV-B species. Among these, we selected the six most frequent types (EV-B: E6, E30, CB5, E5, E9, and CA9). na: not applicable, ns: not significant; WBC: white blood cells.

## DISCUSSION

To our knowledge, this multicentric study assesses for the first time the age-specific cytokine-chemokine response profiles in paired samples of plasma and CSF, with neonates, infants, and children affected with EV-confirmed meningitis being assessed separately. We found that the levels of cytokine/chemokines were upregulated in the CSF of all EV patients, with a fold change higher in children than in neonates, while in plasma, the levels were upregulated in neonates and downregulated in children compared with the controls. This opposite pattern between CSF and plasma likely reflects an age-dependent compartmentalization of the immune response during EV meningitis, with progressively localized cytokine-chemokine production within the central nervous system and tighter regulation of systemic inflammatory responses in older children. The production of cytokine/chemokines in plasma and CSF is modulated by CSF pleocytosis but not the viral load or genotype.

Among patients with EV meningitis, the CSF-to-plasma cytokine-chemokine concentration ratios were higher in children than in infants and neonates, and markedly elevated CSF-to-plasma ratios for several cytokine/chemokines were observed exclusively in children, suggesting a more compartmentalized immune response within their central nervous system. In contrast, CSF cytokine levels and CSF-to-plasma ratios remained low in neonates, which could reflect limited recruitment of peripheral immune cells to the CNS during early life (7). These observations should be confirmed using cytokine indices corrected for blood–brain barrier permeability, such as albumin-adjusted ratios, as previously described (26). In our patients, 32% of the neonates had CSF pleocytosis as against 92% of children. The immune system is relatively immature at birth, and continues to mature throughout the neonatal period and childhood. Monocytes and macrophages of neonates and infants produce a reduced cytokine response compared with that of children and adults (7). Previous studies (7–9) have shown a reduction of 50% in the transmigration of neonatal neutrophils to the infection sites, resulting in increased susceptibility of neonates to bacterial and viral infections. In addition, the immaturity of the central immune response characterized by a low activation of CNS resident immune cells (microglia, astrocytes) results in poor expression of cytokines, immune cell chemotaxis, and activation in infected neonates compared with that in older children (4). Adjustment for time from symptom onset to sampling did not substantially modify the significance of differences in cytokine-chemokine levels between EV meningitis and control patients, except in the CSF of neonates, indicating that sampling time only partially explains the observed differences in cytokine-chemokine responses. In neonates, we showed that CSF is collected earlier after the onset of symptoms than in older children (3), which could explain the lower levels of cytokine/chemokines in the CSF of neonates. The CSF of children, which was sampled 21 h after the onset of symptoms (vs 7 h in neonates and 14 h in infants), had high cytokine-chemokine concentrations, suggesting localized cytokine production rather than passive diffusion, since cytokine-chemokine concentration in the plasma of children was low. The high CSF-to-plasma ratios of IL-8, IL-6, IP-10, and MCP-1 in our EV meningitis children suggest a local synthesis of these proinflammatory cytokine/chemokines. It has been shown that an increase in IL-8 is correlated with EV-A71-infection severity. IL-8, which is especially secreted by macrophages, attracts and activates neutrophils to produce local inflammation (27). Monocytes are activated by the MCP-1 chemokine and release IP-10. High expression of IP-10 in the CNS is crucial to initiate and maintain a protective Th1 response (20).

Severe neurological manifestations are usually associated with cytokine-chemokine storms and trigger the production of high levels of IP-10, IL-8, and IL-17 (28), and high levels of proinflammatory cytokines in general can have adverse effects. In our EV meningitis patients, we observed elevated levels of IP-10, IL-6, and IL-1ra, and moderate levels of IL-8 and INF-γ in CSF. This pattern is indicative of a dynamic balance between the anti-inflammatory and proinflammatory responses. IL-1ra, the natural antagonist against IL-1 cytokines (29), was strongly upregulated in the plasma of our neonates and the CSF of our EV patients. The anti-inflammatory cytokine IL-10 was upregulated in the CSF of our infants and children but not in EV neonates. IL-10 inhibits proinflammatory cytokines, such as IFN-γ, and has a protective role in viral encephalitis. In contrast, an IL-10 polymorphism has been associated with the severity of EV-A71 infections (14, 27). IL-17, which can initiate potent proinflammatory responses (27), was moderately activated in our patients. The relevant comparisons available in the literature concern meningitis or encephalitis caused by EV. Xu et al. reported no difference in cytokine-chemokine expression between patients aged under and over two months affected with EV encephalitis (14). In contrast, we observed a differential cytokine-chemokine expression in the CSF of neonates, infants, and children with EV meningitis. In this previous study, all patients had CSF pleocytosis associated with EV-A71 encephalitis, and we showed that CSF cytokine-chemokine levels increased in the presence of pleocytosis. Cytokine-chemokine response during EV-A71 meningitis was studied in children, or infants and children together, and showed elevated CSF levels of IL-1β, IL-6, IP-10, and IL-8 compared with those in patients with mild hand, foot, and mouth disease (16, 21). We report, for the first time, cytokine-chemokine overexpression specifically in the CSF of neonates.

In the plasma of our EV neonates, the levels of six cytokine/chemokines were significantly increased (IL-1ra, IL-5, IL-6, IFN-γ, IP-10, and MCP-1), and those of MCP-1 and IP-10 were at least 10 times higher than those in the control patients. To our knowledge, the dysregulation of IP-10 and MCP-1 has not been evidenced in the blood of neonates with EV meningitis or encephalitis. IP-10 and MCP-1 are potential biomarkers of other infectious diseases, such as COVID-19, dengue virus, and tuberculosis (27, 30, 31), and may warrant further investigation as potential blood biomarkers for EV infections in neonates that have potentially severe/fatal outcomes.

We also analyzed the influence of factors such as viral load, EV genotype, and pleocytosis on cytokine-chemokine expression in both biological compartments. No association was observed between viral load or EV genotype and cytokine-chemokine expression. We found high levels of CSF cytokine/chemokines (MIP-1β, IL-8, IP-10, IL-1β, IL-6, IFN-γ, TNF-α, IL-1ra, IL-2, and IL-5) in our EV meningitis patients with pleocytosis. The high number of leukocytes in the CSF of neonates (median number of 795 WBC/mm^3^, Table S5) could explain the high levels of cytokine/chemokines in their CSF. In comparison, children had 110 WBC/mm^3^ and lower cytokine-chemokine concentrations in the presence of pleocytosis. These findings suggest that infiltrating WBCs are likely contributors to cytokine-chemokine production in the CSF, although further mechanistic studies will be required to identify the precise cellular sources of cytokine-chemokine production during EV meningitis. Park et al. observed similar patterns of elevated CSF levels of IL-6, IL-8, IFN-γ, TNF-α, and IL-2 in EV meningitis children (mean age 5.5 years) with pleocytosis (13). Migration of immune cells into the CNS was associated with high levels of IL-1β, IL-1ra, IL-6, IL-8, TNF-α, and IFN-γ in the CSF of patients with EV meningitis (32). Data from another study on EV meningitis included a bias by comparing infants without pleocytosis and children with pleocytosis (12). We show that cytokine-chemokine levels were also upregulated in the CSF of neonates and infants with pleocytosis and provide evidence that MCP-1 levels remain unchanged in neonates, infants, and children with pleocytosis. This suggests that CSF MCP-1 could be produced by resident immune cells, independently of peripheral leukocyte influx. In plasma, MCP-1, IP-10, IL-6, INF-γ, IL-1ra, and IL-5 were upregulated in EV neonates and infants in the absence of CSF pleocytosis, suggesting that the inflammatory response is predominantly located in the bloodstream during EV meningitis. In contrast, cytokine-chemokine levels were stable in the plasma of children with and without CSF pleocytosis, possibly owing to lower activation of the cytokine-chemokine response due to immune system maturation in children.

There are several limitations to our study. First, the controls were not healthy individuals, and most had fever of unknown origin, particularly the very young infants, or viral respiratory infection. However, several cytokine/chemokines, especially IFNγ, were not detected in these patients, suggesting that they are suitable controls. Second, the number of subjects in the neonate group was smaller than that of older children, and there were only five children without CSF pleocytosis in our cohort. Third, sensitivity analyses restricted to PCR-confirmed EV meningitis in the CSF revealed cytokine-chemokine expression patterns that were similar to those observed in the overall cohort. Seven additional cytokine/chemokines became significant in the CSF of neonates when all four criteria (fold change, effect size, ROC, and *P*-value) were considered in the patients with a positive EV PCR result in the CSF. The plasma and CSF samples were obtained from pediatric patients in the acute phase of EV meningitis. The levels of cytokine/chemokines detected in this study are only suitable for use as acute biomarkers. Our study also has its strengths: the age of patients was well matched across the EV and control groups; pleocytosis, EV genotype, and viral load were assessed; and cytokine-chemokine levels were determined by two measurements made in accordance with the manufacturer’s recommendations.

In conclusion, our study clearly shows that it is misleading to interpret cytokine-chemokine levels in EV meningitis in children aged 0–16 years without separating patients into the age categories of neonates, infants, and children since the expression of cytokine/chemokines varies with patient age. In CSF, the secretion of cytokine/chemokines in EV patients is low in neonates and very high in children compared with the respective control patients. In contrast, overexpression of cytokine/chemokines was observed in the plasma of neonates compared with that of children. Our study gives an overview of the cytokine-chemokine signature profiles observed in EV meningitis. It establishes a descriptive, age-stratified framework for future studies investigating immune responses in severe EV-associated neurological infections, in which cytokines and chemokines could be evaluated as potential biomarkers, particularly in neonates who are at higher risk of severe disease.

## Data Availability

All data that support the findings of this study are included in the article and its supplemental material.

## SUPPLEMENTAL MATERIAL

Supplemental material jvi.01871-25-s0001.docx Supplemental methods, Tables S1 to S5, and Fig. S1.

ASM does not own the copyrights to Supplemental Material that may be linked to, or accessed through, an article. The authors have granted ASM a non-exclusive, world-wide license to publish the Supplemental Material files. Please contact the corresponding author directly for reuse.
