# Supplementary material for: Differential cytokine signature profiles in neonates, infants, and children with enterovirus meningitis
Source: J Virol. 2026 May 11;100(6):e01871-25. doi: 10.1128/jvi.01871-25 (PMC13288476; doi:10.1128/jvi.01871-25)
Supplement: Supplemental material — Supplemental methods, Tables S1 to S5, and Fig. S1. [file jvi.01871-25-s0001.docx]

**Supplemental Material**

**Differential cytokine signature profiles in neonates, infants and children with enterovirus meningitis**

**Contents**

[Supplementary methods 2](#_Toc200036496)

Serum and CSF samples stored at -80°C were thawed and centrifuged at 1000xg for 15 min at 4°C.

[Statistical analysis 2](#_Toc200036498)

[Supplementary Tables and Figures 4](#_Toc200036499)

[Table S1: Cytokines and chemokines included in the multiplex bead assay. 4](#_Toc200036500)

[Table S2: Statistical analysis of the difference of cytokine-chemokine expression between control neonates, infants and children, and between EV neonates, infants and children, in plasma and CSF 5](#_Toc200036501)

[Table S3: Cytokine-chemokine expression in neonates, infants and children with EV meningitis and controls, in the plasma and the CSF 7](#_Toc200036502)

[Table S4: Expression of cytokines and chemokines in neonates, infants and children with EV meningitis (restricted to PCR-positive results for EV in the CSF) compared with control patients. 11](#_Toc200036502)

[Figure S1: Venn diagram showing the 12 cytokine/chemokines significantly over-expressed](#_Toc200036503) shared between EV age groups 15

[Table S5: Characteristics of EV neonates, infants and children with and without pleocytosis 16](#_Toc200036502)

# Supplemental methods

## Cytokine-chemokine analysis by the multiplex bead assay

The magnetic bead-based multiplex immunoassay Bio-Plex^®^ Pro Human Cytokine Panel 27-plex assay (Bio-Rad, Hercules, CA) was performed according to the manufacturer’s instructions. Serum and CSF samples stored at -80°C were thawed and centrifuged at 1000xg for 15 min at 4°C. Plasma and CSF samples were diluted 1:4 and 1:2, respectively (diluent was the sample diluent HB provided in the assay kit), and were tested in duplicate. Fluorescent signals were acquired on a validated and calibrated Bio-Plex 200 system (Bio-Rad) and data were analysed using the Bio-Plex Manager Software 6.1 (Bio-Rad). Signals lower than the minimum detectable concentration were attributed the limit of detection (LOD) minus one unit for analysis; i.e. LOD [IL-1β] = 0.8 pg/mL, if sample value was < 0.8, the sample was attributed 0.7 pg/mL.

## Statistical analysis

Patient characteristics were expressed as the median [interquartile range] for continuous data. The assumption of normality was assessed by the Shapiro-Wilk test. Continuous variables (such as cytokines-chemokines) were compared between independent groups (such as EV positive patients *vs* their controls, neonates *vs* infants *vs* children, EV with pleocytosis *vs* EV without pleocytosis *vs* controls) by analysis of variance (ANOVA) or Kruskal-Wallis test if the conditions of ANOVA were not met (normality and homoscedasticity verified by the Bartlett test). When appropriate (omnibus p-value less than 0.05), post-hoc tests for two-by-two multiple comparisons were applied: Tukey-Kramer after ANOVA and Dunn after Kruskal-Wallis test. The results were expressed using fold change, effect size and ROC (Receiver Operating Characteristic) area curve. The comparisons between groups were performed by Chi-square test or when appropriate by Fisher’s exact test for categorical variables. In addition, the representativeness of EV patients without EV RNA detected in the CSF (2 neonates, 7 infants, and 3 children) was analysed with the univariate statistical tests cited above. The analyses were conducted separately for newborns, infants and children. The study of the relationships between quantitative variables was performed using correlation coefficients (Pearson or Spearman according to statistical distribution). For comparisons of EV meningitis and control patients, a multivariate analysis adjusted for sample collection time was performed. For comparisons of EV types, multivariate analyses were conducted to take into account possible confounders, they were adjusted for age, sample time, viral load, and pleocytosis for CSF, or were adjusted for age, sample time, viral load, and WBC for plasma. Finally, for paired comparisons (*i.e.* viral load in CSF and in blood), Wilcoxon test was used. Statistical analyses concerning the comparisons of cytokines-chemokines were conducted for all age groups (neonates, infants and children). Analyses were performed with Stata software (version 15, StataCorp, College Station, TX). Two-sided tests with α = 0.05 were used. Sidak’s correction was applied to take into account multiple comparisons. The results were also expressed using effect-sizes (ES) and 95% confidence intervals and were interpreted according to the recommendations of Cohen, who defined the ES bounds as small (ES = 0.2), medium (ES = 0.5), and large (ES = 0.8). For the analysis of CSF/plasma ratios of cytokine-chemokine concentrations, we included samples from patients if the time between the venepuncture and the lumbar puncture was less than eight hours and if the red blood cell count in the CSF was less than 160/mm³. For the comparison of cytokine-chemokine expression in patients with or without pleocytosis, we included samples from patients if the red blood cell count in the CSF was less than 160/mm³.

# Supplemental Tables and Figures

### **Table S1**

### **Cytokines** and chemokines included in the multiplex bead assay.

|  | Cytokine | *Alternative name* | Minimum detectable concentration (pg/mL) |
| --- | --- | --- | --- |
| Chemoattractant | |  |  |
|  | MCP-1 | *CCL2* | 6.7 |
|  | MIP-1a | *CCL3* | 2.4 |
|  | MIP-1b | *CCL4* | 1.1 |
|  | Eotaxin | *CCL11* | 14.6 |
| *Endothelial activation, cerebral transmigration during inflammation* | | | |
|  | IL-8* | *CXCL8* | 0.5 |
|  | IP-10 | *CXCL10* | 6.5 |
|  | RANTES | *CCL5* | 1.2 |
| Pro-inflammatory response (Th1 profile) | | |  |
|  | IL-1β | *catabolin* | 0.8 |
|  | IL-6 | *BSF-2, CDF* | 1.1 |
|  | IFN-γ | - | 19.3 |
|  | TNF-α | *cachectin* | 3.0 |
| Super pro-inflammatory response (Th17 profile) | | |  |
|  | IL-17a | *CTLA-8* | 0.2 |
| Anti-inflammatory response (Th2 profile) | | |  |
|  | IL-1ra | *ICIL-1RA* | 1.4 |
|  | IL-4▪ | *BSF-1* | 0.5 |
|  | IL-10 | *CSIF* | 0.9 |
|  | IL-13▪ | *-* | 2.1 |
| Adaptive immunity and lymphocyte activation | | |  |
|  | IL-2 | *TCGF* | 1.1 |
|  | IL-5 | *B-cell /eosinophil differentiation factor; TRF* | 0.8 |
|  | IL-7 | *-* | 0.5 |
|  | IL-9 | *Cytokine P40; T-cell growth factor P40* | 0.7 |
|  | IL-12 | *NKSF ; CLMF p35/40* | 0.5 |
|  | IL-15 | *-* | 4.2 |
| Growth factor | |  |  |
|  | G-CSF | *-* | 1.1 |
|  | GM-CSF | *CSF* | 4.5 |
|  | VEGF | *VPF* | 0.5 |
|  | FGF-basic | *FGF-2 ; HBGF-2* | 6.8 |
|  | PDGF-bb | *PDGF-2* | 1.0 |

*IL-8 is a chemoattractant also involved in the pro-inflammatory response; ▪ IL-4 and 13 are involved in the anti-inflammatory response and the adaptive immunity.

## **Table S2**

## **Statistical analysis of the difference of cytokine-chemokine expression between control neonates, infants and children, and between EV neonates, infants and children, in plasma and CSF.**

|  | **Control patients** | | | | | | |
| --- | --- | --- | --- | --- | --- | --- | --- |
|  | Cytokine concentration control neonates (n=10) | Cytokine concentration control infants (n=15) | cytokine concentration control children (n=30) | *p value* | neonates *vs* infants | children *vs* neonates | children *vs* infants |
| **PLASMA** |  |  |  |  |  |  |  |
| MCP-1 | 46.92 [29.99-54.74] | 36.36 [19.04-83.67] | 31.21 [18.85-67.94] | *0.657* | - | - | - |
| MIP-1a | 2.70 [2.30-3.93] | 2.87 [2.30-3.90] | 2.30 [2.30-2.92] | *0.657* | - | - | - |
| MIP-1b | 220.20 [206.13-241.85] | 229.17 [220.95-242.77] | 225.16 [194.49-243.28] | *0.152* | - | - | - |
| Eotaxin | 41.61 [34.86-51.14] | 34.92 [25.31-49.64] | 31.52 [21.09-41.75] | *0.769* | - | - | - |
| IL-8 | 14.39 [8.06-19.05] | 14.98 [10.51-23.92] | 6.37 [4.24-13.86] | *0.172* | - | - | - |
| IP-10 | 610.70 [255.07-3543.98] | 879.19 [274.72-2861.01] | 1277.03 [392.72-2621.44] | *0.014* | 0.347 | 0.585 | 0.589 |
| RANTES | 5348.81 [4423.32-7643.51] | 4751.41 [4375.80- 7278.03] | 6936.32 [4470.84-8839.52] | *0.861* | - | - | - |
| IL-1β | 3.07 [2.67-4.24] | 3.3 [2.92-4.61] | 3.10 [2.55-4.93] | *0.444* | - | - | - |
| IL-6 | 3.92 [2.74-8.06] | 4.49 [3.24-10.09] | 9.67 [2.58-21.58] | *0.837* | - | - | - |
| IFN-γ | 19.20 [19.20-20.45] | 19.20 [19.20-19.20] | 19.20 [19.20-24.03] | *0.105* | - | - | - |
| TNF-α | 83.22 [64.08-98.60] | 87.88 [68.64-102.19] | 73.22 [61.05-91.60] | *0.136* | - | - | - |
| IL-17 | 17.64 [16.33-20.48] | 19.71 [17.02-25.6] | 15.21 [13.26-17.89] | *0.448* | - | - | - |
| IL-1ra | 473.31 [114.05-2002.55] | 780.67 [249.34-1470.93] | 1217.32 [597.55-3204.93] | *0.023* | 0.632 | 0.999 | 0.881 |
| IL-4 | 4.55 [4.08-5.05] | 4.61 [3.44-6.22] | 4.23 [3.29-5.04] | *0.136* | - | - | - |
| IL-10 | 3.09 [0.80-7.15] | 4.47 [0.80-14.31] | 5.08 [1.32-15.29] | *0.402* | - | - | - |
| IL-13 | 2.12 [2.00-2.40] | 2.22 [2.00-3.14] | 2.15 [2.00-3.01] | *0.504* | - | - | - |
| IL-2 | 4.32 [2.73-6.77] | 7.25 [3.36-8.57] | 3.83 [2.28-8.13] | *0.850* | - | - | - |
| IL-5 | 40.62 [14.38-58.32] | 34.91 [21.68-49.63] | 35.80 [13.21-67.29] | *0.476* | - | - | - |
| IL-7 | 27.94 [24.36-35.05] | 24.18 [21.24-36.18] | 21.59 [19.10-29.34] | *0.917* | - | - | - |
| IL-9 | 310.68 [277.42-346.29] | 323.93 [302.13-354.13] | 312.55 [269.55-347.46] | *0.300* | - | - | - |
| IL-12 | 4.17 [1.31-6.09] | 6.64 [5.03-11.75] | 4.89 [4.18-13.43] | *0.709* | - | - | - |
| IL-15 | 4.10 [4.10-4.10] | 4.10 [4.10-4.10] | 4.10 [4.10-200.74] | *0.049* | 0.012 | 0.065 | 0.697 |
| G-CSF | 148.74 [134.28-239.51] | 148.09 [136.87-210.62] | 125.16 [103.37-181.47] | *0.594* | - | - | - |
| GM-CSF | 4.40 [4.40-4.87] | 4.40 [4.40-5.08] | 4.40 [4.40-6.40] | *0.067* | - | - | - |
| VEGF | 0.40 [0.40-3.22] | 0.40 [0.40-0.40] | 0.40 [0.40-201.57] | *0.805* | - | - | - |
| FGF-basic | 58.40 [53.51-64.96] | 76.53 [64.26-86.88] | 64.97 [54.75-86.99] | *0.318* | - | - | - |
| PDGF-bb | 445.51 [307.12-1070.37] | 758.76 [568.83 -939.57] | 724.76 [371.72-1180.90] | *0.084* | - | - | - |
| **CEREBROSPINAL FLUID** | |  |  |  |  |  |  |
| MCP-1 | 722.41 [571.47-847.90] | 511.785 [428.12-604.35] | 352.79 [153.05-1055.16] | *0.067* | - | - | - |
| MIP-1a | 2.30 [2.30-2.30] | 2.30 [2.30-2.30] | 2.30 [2.30-2.30] | *0.010* | 0.336 | 0.336 | na |
| MIP-1b | 11.17 [6.36-17.89] | 6.02 [3.69-9.97] | 6.73 [3.98-9.85] | *0.066* | - | - | - |
| Eotaxin | - | - | - | *-* | - | - | - |
| IL-8 | 66.37 [53.95-109.32] | 52.33 [34.96-70.71] | 75.16 [37.14-105.69] | *0.249* | - | - | - |
| IP-10 | 688.90 [189.34-1177.58] | 188.03 [108.9-644.34] | 358.26 [112.18-1594.00] | *0.423* | - | - | - |
| RANTES | 6.72 [6.09-73.11] | 11.46 [6.35-12.83] | 11.77 [8.25-13.95] | *0.502* | - | - | - |
| IL-1β | 1.13 [1.01-1.22] | 0.85 [0.70-1.36] | 0.70 [0.70-0.98] | *0.089* | - | - | - |
| IL-6 | 1.00 [1.00-1.00] | 1.00 [1.00-1.00] | 5.91 [2.42-13.81] | *< 0.001* | 0.521 | 0.08 | 0.082 |
| IFN-γ | 19.20 [19.20-20.12] | 19.20 [19.20-19.20] | 19.20 [19.20-21.36] | *0.043* | 0.346 | 0.342 | 0.044 |
| TNF-α | 6.77 [3.05-8.94] | 2.90 [2.90-2.90] | 2.90 [2.90-4.26] | *0.003* | 0.023 | 0.083 | 0.104 |
| IL-17 | 1.58 [1.00-1.95] | 1.11 [0.66-2.20] | 1.08 [0.66-1.70] | *0.666* | - | - | - |
| IL-1ra | 110.94 [1.30-156.98] | 49.85 [1.30-151.64] | 96.60 [54.60-179.75] | *0.335* | - | - | - |
| IL-4 | 0.40 [0.40-0.40] | 0.40 [0.40-0.54] | 0.44 [0.40-0.52] | *0.149* | - | - | - |
| IL-10 | 0.80 [0.80-0.80] | 0.80 [0.80-2.29] | 3.06 [2.26-3.75] | *< 0.001* | 0.117 | < 0.001 | 0.001 |
| IL-13 | 2.00 [2.00-2.00] | 2.00 [2.17-2.37] | 2.00 [2.00-2.18] | *0.196* | - | - | - |
| IL-2 | 1.00 [1.00-1.52] | 1.00 [1.00-1.00] | 1.16 [1.00-1.73] | *0.061* | - | - | - |
| IL-5 | 13.12 [3.74-20.77] | 14.42 [9.10-18.75] | 30.11 [20.77-35.21] | *< 0.001* | 0.882 | < 0.001 | 0.001 |
| IL-7 | 5.38 [1.51-6.03] | 1.51 [0.40-2.20] | 0.40 [0.40-0.40] | *< 0.001* | 0.084 | 0.011 | 0.13 |
| IL-9 | 3.90 [1.84-24.39] | 3.84 [0.60-5.37] | 7.54 [4.74-9.08] | *0.006* | 0.269 | 0.622 | < 0.001 |
| IL-12 | 0.91 [0.40-1.31] | 0.90 [0.40-1.81] | 1.53 [0.40-2.02] | *0.535* | - | - | - |
| IL-15 | 4.10 [4.10-4.10] | 4.10 [4.10-4.10] | 56.85 [4.10-121.16] | *< 0.001* | 0.589 | < 0.001 | 0.001 |
| G-CSF | 41.33 [29.69-68.25] | 32.14 [20.88-86.04] | 49.53 [35.57-92.12] | *0.503* | - | - | - |
| GM-CSF | - | - | - | *-* | - | - | - |
| VEGF | 0.40 [0.40-0.40] | 0.40 [0.40-0.40] | 61.96 [39.14-99.60] | *< 0.001* | 0.589 | < 0.001 | < 0.001 |
| FGF-basic | 9.86 [6.70-16.75] | 6.70 [6.70-8.99] | 8.67 [6.70-12.13] | *0.138* | - | - | - |
| PDGF-bb | 9.90 [6.24-15.85] | 23.30 [10.49-40.67] | 25.94 [18.92-38.45] | *< 0.001* | 0.018 | < 0.001 | 0.838 |
|  |  |  |  |  |  |  |  |
|  | **EV patients** | | | | | | |
|  | Cytokine concentration EV neonates (n=19) | Cytokine concentration EV infants (n=29) | cytokine concentration EV children (n=60) | *p value* | neonates *vs* infants | children *vs* neonates | children *vs* infants |
| **PLASMA** |  |  |  |  |  |  |  |
| MCP-1 | 487.96 [83.85- 701.94] | 146.95 [35.06-313.37] | 11.05 [8.27-17.95] | *< 0.001* | 0.053 | < 0.001 | < 0.001 |
| MIP-1a | 5.436 [3.39-6.12] | 3.39 [2.30-4.34] | 2.30 [2.30-2.30] | *< 0.001* | 0.027 | < 0.001 | < 0.001 |
| MIP-1b | 221.87 [210.87-256.04] | 226.92 [203.05-236.42] | 204.44 [187.70-223.03] | *0.005* | 0.903 | 0.058 | 0.195 |
| Eotaxin | 36.12 [26.91-53.61] | 28.04 [22.03-35.87] | 16.28 [14.50-23.22] | *< 0.001* | 0.125 | 0.004 | 0.001 |
| IL-8 | 23.76 [19.05-28.48] | 13.22 [8.92-25.34] | 5.22 [3.16-8.40] | *< 0.001* | 0.458 | 0.066 | 0.032 |
| IP-10 | 6148.58 [3583.49-8952.03] | 3670.8 [1645.98-6487.98] | 460.21 [302.13-640.54] | *< 0.001* | 0.198 | < 0.001 | < 0.001 |
| RANTES | 4586.48 [4362.6-6333.31] | 5170.96 [4316.74-6580.58] | 5027.81 [3856.10-6170.10] | *0.520* | - | - | - |
| IL-1β | 4.7 [3.4-5.94] | 3.38 [2.67-5.07] | 2.23 [1.82-2.83] | *< 0.001* | 0.208 | 0.021 | 0.062 |
| IL-6 | 24.84 [12.71-66.1] | 8.59 [2.50-25.38] | 6.48 [3.37-15.22] | *< 0.001* | 0.06 | 0.009 | 0.352 |
| IFN-γ | 73.71 [26.18-113.37] | 22.24 [19.20-49.39] | 19.20 [19.20-19.20] | *< 0.001* | 0.014 | 0.001 | 0.004 |
| TNF-α | 88.38 [73.19-95.52] | 79.31 [63.63-88.25] | 51.75 [46.01-59.65] | *< 0.001* | 0.773 | < 0.001 | 0.001 |
| IL-17 | 20.29 [15.22-23.13] | 16.74 [12.94-20.14] | 12.38 [10.76-14.26] | *< 0.001* | 0.206 | < 0.001 | 0.001 |
| IL-1ra | 4605.04 [3417.92-6322.305] | 1790.42 [699.37-4273.64] | 466.94 [195.80-873.00] | *< 0.001* | 0.041 | < 0.001 | < 0.001 |
| IL-4 | 4.62 [3.19-5.83] | 3.47 [2.46-4.35] | 2.43 [2.02-3.17] | *< 0.001* | 0.096 | 0.001 | 0.013 |
| IL-10 | 2.11 [0.80-3.89] | 1.55 [0.80-3.18] | 0.80 [0.80-1.08] | *< 0.001* | 0.659 | 0.244 | 0.05 |
| IL-13 | 2.00 [2.00-2.49] | 2.00 [2.00-2.12] | 2.00 [2.00-2.44] | *0.8971* | - | - | - |
| IL-2 | 8.76 [6.43-10.71] | 5.12 [1.63-7.69] | 2.04 [1.00-3.95] | *< 0.001* | 0.003 | < 0.001 | 0.022 |
| IL-5 | 93.19 [53.59-115.96] | 43.69 [26.48-73.27] | 22.34 [8.71-42.04] | *< 0.001* | 0.006 | < 0.001 | 0.342 |
| IL-7 | 27.95 [22.52_31.24] | 22.05 [16.23-28.28] | 17.23 [12.78-22.47] | *< 0.001* | 0.209 | 0.089 | 0.692 |
| IL-9 | 296.29 [233.0-352.61] | 304.98 [247.08-333.72] | 285.62 [246.26-322.88] | *0.601* | - | - | - |
| IL-12 | 8.29 [2.31-9.63] | 2.89 [1.72-7.54] | 3.70 [1.61-8.34] | *0.189* | - | - | - |
| IL-15 | 4.10 [4.10-4.10] | 4.10 [4.10-4.10] | 4.10 [4.10-4.10] | *0.348* | - | - | - |
| G-CSF | 237.08 [188.44 -296.57] | 168.51 [132.47-216.18] | 82.17 [63.76-105.17] | *< 0.001* | 0.018 | < 0.001 | < 0.001 |
| GM-CSF | 5.61 [4.4-7.34] | 4.40 [4.40-6.19] | 4.40 [4.40-5.23] | *0.019* | 0.509 | 0.488 | 1,000 |
| VEGF | 0.40 [0.40-63.01] | 0.40 [0.40-0.40] | 0.40 [0.40-0.40] | *0.122* | - | - | - |
| FGF-basic | 80.10 [67.5-90.74] | 63.97 [52.84-81.41] | 57.38 [46.59-66.17] | *< 0.001* | 0.106 | < 0.001 | 0.061 |
| PDGF-bb | 501.31 [295.67-809.83] | 608.42 [340.25- 1075.56] | 692.68 [413.65-1192.36] | *0.229* | - | - | - |
| **CEREBROSPINAL FLUID** | |  |  |  |  |  |  |
| MCP-1 | 957.37 [742.98-1572.63] | 1245.04 [740.59 - 1646.55] | 1309.94 [674.11-2085.58] | *0.756* | - | - | - |
| MIP-1a | 2.30 [2.30-39.93] | 5.61 [2.30-17.74] | 5.59 [3.67-9.26] | *0.845* | - | - | - |
| MIP-1b | 34.02 [10.9-195.96] | 54.46 [23.07-164.91] | 48.77 [34.87-103.63] | *0.663* | - | - | - |
| Eotaxin | - | - | - | *-* | - | - | - |
| IL-8 | 222.79 [102.33-3205.6] | 426.59 [149.91-1170.07] | 1946.39 [1013.15-5576.32] | *< 0.001* | 0.612 | 0.004 | < 0.001 |
| IP-10 | 8806.23 [310.21-15865.82] | 7743.59 [2252.69-13434.49] | 18701.94 [14508.30-26808.50] | *< 0.001* | 0.844 | < 0.001 | < 0.001 |
| RANTES | 13.2 [5.42-79.89] | 29.02 [10.81-122.65] | 26.80 [21.12-43.32] | *0.154* | - | - | - |
| IL-1β | 3.44 [0.90-19.75] | 3.96 [1.73-8.74] | 7.40 [5.96-15.86] | *0.0046* | 0.306 | 0.854 | 0.016 |
| IL-6 | 10. 67 [1-1786] | 53.06 [1.87-364.71] | 990.74 [446.73-2251.23] | *< 0.001* | 0.594 | 0.012 | < 0.001 |
| IFN-γ | 63.83 [19.20-129.12] | 60.71 [19.20-97.40] | 77.02 [47.95-151.24] | *0.103* | - | - | - |
| TNF-α | 13.75 [8.61-73.32] | 20.49 [10.04-38.98] | 34.93 [23.55-49.72] | *0.053* | - | - | - |
| IL-17 | 4.15 [0.10-19.6] | 6.72 [2.94-13.92] | 13.25 [9.08-18.43] | *< 0.001* | 0.94 | 0.057 | < 0.001 |
| IL-1ra | 595.75 [194.5-9849.9] | 1020.14 [281.24-4955. 23] | 6640.44 [3487.17-8690.14] | *< 0.001* | 0.628 | 0.899 | 0.012 |
| IL-4 | 0.61 [0.40-3.28] | 1.12 [0.51-2.1] | 2.44 [1.70-3.69] | *< 0.001* | 0.849 | 0.023 | < 0.001 |
| IL-10 | 0.80 [0.80-10.21] | 3.99 [0.81-7.04] | 28.23 [14.61-44.43] | *< 0.001* | 0.997 | < 0.001 | < 0.001 |
| IL-13 | 2.00 [2.00-2.00] | 2.00 [2.00-2.00] | 2.00 [2.00-2.06] | *0.418* | - | - | - |
| IL-2 | 3.88 [1.00-17.31] | 4.7 [2.04-9.24] | 9.44 [7.06-12.70] | *< 0.001* | 0.668 | 0.211 | < 0.001 |
| IL-5 | 36.62 [12.78-185.23] | 58.78 [27.08-98.07] | 161.84 [103.98-203.34] | *< 0.001* | 0.843 | 0.009 | < 0.001 |
| IL-7 | 6.77 [4.82-.35] | 6.34 [2.15-9.48] | 7.67 [4.34-14.30] | *0.239* | - | - | - |
| IL-9 | 16.86 [1.8-60.44] | 21.1 [8.13-88.32] | 50.22 [33.06-75.09] | *0.0034* | 0.276 | 0.007 | 0.628 |
| IL-12 | 1.7 [0.40-7.96] | 2.44 [0.90-4.36] | 5.53 [4.10-8.83] | *< 0.001* | 0.618 | 0.261 | < 0.001 |
| IL-15 | 4.10 [4.10-47.22] | 4.10 [4.10-28.12] | 115.27 [4.10-186.36] | *< 0.001* | 0.998 | 0.003 | 0.001 |
| G-CSF | 102.33 [35.98-1810.32] | 200.71 [99.87-873.09] | 383.74 [185.33-730.70] | *0.189* | - | - | - |
| GM-CSF | - | - | - | *-* | - | - | - |
| VEGF | 0.40 [0.40-86.07] | 22.88 [0.40-85.71] | 131.69 [91.79-150.55] | *< 0.001* | 0.848 | < 0.001 | < 0.001 |
| FGF-basic | 43.60 [8.72-80.69] | 44.38 [19.2-67.61] | 59.52 [50.16-76.03] | *0.045* | 0.924 | 0.575 | 0.033 |
| PDGF-bb | 18.00 [7.48-80.34] | 40.02 [16.83-85.05] | 70.28 [52.92-104.66] | *< 0.001* | 0.369 | 0.008 | 0.321 |

Cytokine-chemokine expression (pg/mL) is expressed in median [IQR] for EV (EV meningitis) and control groups.

**Table S3.** Expression of cytokines and chemokines in the plasma and CSF of neonates, infants and children with EV meningitis and controls.

|  |  | | **NEONATES** | | | | | | |
| --- | --- | --- | --- | --- | --- | --- | --- | --- | --- |
|  |  | | Cytokine concentration  EV meningitis (n=19) | Cytokine concentration Control (n=10) | | Fold Change | Effect Size | ROC | *p value* |
| **PLASMA** | | |  |  | |  |  |  |  |
| Chemokines | | |  |  | |  |  |  |  |
|  | MCP-1 | | 487.96 [83.85- 701.94] | 46.92 [29.99-54.74] | | 10.40 | 1.22 | 0.84 | *0.003* |
|  | MIP-1a | | 5.436 [ 3.39-6.12] | 2.70 [2.30-3.93] | | 2.01 | 1.25 | 0.85 | *0.002** |
|  | MIP-1b | | 221.87 [210.87-256.04] | 220.20 [206.13-241.85] | | 1.00 | 0.07 | 0.55 | *0.679* |
|  | Eotaxin | | 36.12 [26.91-53.61] | 41.61 [34.86-51.14] | | -1.15 | -0.21 | 0.42 | *0.463* |
|  | IL-8 | | 23.76 [19.05-28.48] | 14.39 [8.06-19.05] | | 1.65 | 0.97 | 0.82 | *0.005** |
|  | IP-10 | | 6148.58 [3583.49-8952.03] | 610.70 [255.07-3543.98] | | 10.07 | 1.48 | 0.80 | *0.009** |
|  | RANTES | | 4586.48 [ 4362.6-6333.31] | 5348.81 [4423.32-7643.51] | | -1.17 | -0.03 | 0.46 | *0.748* |
| Pro-inflammatory response | | | |  | |  |  |  |  |
|  | IL-1β | | 4.7 [3.4-5.94] | 3.07 [2.67-4.24] | | 1.53 | 0.71 | 0.71 | *0.075* |
|  | IL-6 | | 24.84 [12.71-66.1] | 3.92 [2.74-8.06] | | 6.34 | 1.73 | 0.93 | *< 0.001* |
|  | IFN-γ | | 73.71 [26.18-113.37] | 19.20 [19.20-20.45] | | 3.84 | 1.36 | 0.85 | *0.002** |
|  | TNF-α | | 88.38 [73.19-95.52] | 83.22 [64.08-98.60] | | 1.06 | 0.19 | 0.55 | *0.646* |
|  | IL-17 | | 20.29 [15.22-23.13] | 17.64 [16.33-20.48] | | 1.15 | 0.09 | 0.57 | *0.566* |
| Anti-inflammatory response | | | |  | |  |  |  |  |
|  | IL-1ra | | 4605.04 [3417.92-6322.305] | 473.31 [114.05-2002.55] | | 9.73 | 1.61 | 0.89 | *< 0.001** |
|  | IL-4 | | 4.62 [3.19-5.83] | 4.55 [4.08-5.05] | | 1.02 | -0.008 | 0.51 | *0.909* |
|  | IL-10 | | 2.11 [0.80-3.89] | 3.09 [0.80-7.15] | | -1.46 | -0.34 | 0.41 | *0.415* |
|  | IL-13 | | 2.00 [2.00-2.49] | 2.12 [2.00-2.40] | | -1.06 | -0.05 | 0.44 | *0.570* |
| Adaptive immunity and lymphocyte activation | | | |  | |  |  |  |  |
|  | IL-2 | | 8.76 [6.43-10.71] | 4.32 [2.73-6.77] | | 2.03 | 1.22 | 0.82 | *0.006** |
|  | IL-5 | | 93.19 [53.59-115.96] | 40.62 [14.38-58.32] | | 2.3 | 1.16 | 0.79 | *0.010** |
|  | IL-7 | | 27.95 [22.52_31.24] | 27.94 [24.36-35.05] | | 1.00 | -0.39 | 0.42 | *0.491* |
|  | IL-9 | | 296.29 [233.0-352.61] | 310.68 [277.42-346.29] | | -1.05 | -0.24 | 0.44 | *0.582* |
|  | IL-12 | | 8.29 [2.31-9.63] | 4.17 [1.31-6.09] | | 1.99 | 0.65 | 0.71 | *0.066* |
|  | IL-15 | | 4.10 [4.10-4.10] | 4.10 [4.10-4.10] | | 1.00 | 0.20 | 0.52 | *0.795* |
| Growth factors | | |  |  | |  |  |  |  |
|  | G-CSF | | 237.08 [188.44 -296.57] | 148.74 [134.28-239.51] | | 1.59 | 0.87 | 0.74 | *0.035** |
|  | GM-CSF | | 5.61 [4.4-7.34] | 4.40 [4.40-4.87] | | 1.28 | 0.76 | 0.73 | *0.034* |
|  | VEGF | | 0.40 [0.40-63.01] | 0.40 [0.40-3.22] | | 1,00 | 0.12 | 0.50 | *0.978* |
|  | FGF-basic | | 80.10 [67.5-90.74] | 58.40 [53.51-64.96] | | 1.37 | 1.39 | 0.87 | *0.001** |
|  | PDGF-bb | | 501.31 [295.67-809.83] | 445.51 [307.12-1070.37] | | 1.13 | 0.14 | 0.50 | *1.000* |
| **CEREBROSPINAL FLUID** | | | |  | |  |  |  |  |
| Chemokines | | |  |  | |  |  |  |  |
|  | MCP-1 | | 957.37 [742.98-1572.63] | 722.41 [571.47-847.90] | | 1.33 | 0.96 | 0.77 | *0.019* |
|  | MIP-1a | | 2.30 [2.30-39.93] | 2.30 [2.30-2.30] | | 1,00 | 0.83 | 0.68 | *0.066* |
|  | MIP-1b | | 34.02 [10.9-195.96] | 11.17 [6.36-17.89] | | 3.05 | 0.92 | 0.74 | *0.039** |
|  | Eotaxin | | - | - | | - | - | - | *-* |
|  | IL-8 | | 222.79 [102.33-3205.6] | 66.37 [53.95-109.32] | | 3.36 | 1.36 | 0.90 | *< 0.001* |
|  | IP-10 | | 8806.23 [310.21-15865.82] | 688.90 [189.34-1177.58] | | 12.78 | 1.64 | 0.86 | *0.002* |
|  | RANTES | | 13.2 [5.42-79.89] ^¤^ | 6.72 [6.09-73.11] | | 1.96 | 0.12 | 0.51 | *0.927* |
| Pro-inflammatory response | | | |  | |  |  |  |  |
|  | IL-1β | | 3.44 [0.90-19.75] | 1.13 [1.01-1.22] | | 3.04 | 0.96 | 0.70 | *0.081* |
|  | IL-6 | | 10. 67 [1-1786] | 1.00 [1.00-1.00] | | 10.67 | 1.11 | 0.85 | *0.001* |
|  | IFN-γ | | 63.83 [19.20-129.12] | 19.20 [19.20-20.12] | | 3.32 | 1.13 | 0.76 | *0.001* |
|  | TNF-α | | 13.75 [8.61-73.32] | 6.77 [3.05-8.94] | | 2.03 | 1.02 | 0.78 | *0.015* |
|  | IL-17 | | 4.15 [0.10-19.6] | 1.58 [1.00-1.95] | | 2.63 | 0.71 | 0.63 | *0.239* |
| Anti-inflammatory response | | | |  | |  |  |  |  |
|  | IL-1ra | | 595.75 [194.5-9849.9] | 110.94 [1.30-156.98] | | 5.37 | 1.35 | 0.90 | *< 0.001* |
|  | IL-4▪ | | 0.61 [0.40-3.28] | 0.40 [0.40-0.40] | | 1.53 | 1.02 | 0.81 | *0.005* |
|  | IL-10 | | 0.80 [0.80-10.21] | 0.80 [0.80-0.80] | | 1.00 | 0.75 | 0.62 | *0.198* |
|  | IL-13▪ | | 2.00 [2.00-2.00] | 2.00 [2.00-2.00] | | 1.00 | -0.35 | 0.47 | *0.702* |
| Adaptive immunity and lymphocyte activation | | | |  | |  |  |  |  |
|  | IL-2 | | 3.88 [1.00-17.31] | 1.00 [1.00-1.52] | | 3.88 | 0.95 | 0.67 | *0.114* |
|  | IL-5 | | 36.62 [12.78-185.23] | 13.12 [3.74-20.77] | | 2.79 | 1.06 | 0.75 | *0.028* |
|  | IL-7 | | 6.77 [4.82-9.35] | 5.38 [1.51-6.03] | | 1.26 | 0.58 | 0.66 | *0.154* |
|  | IL-9 | | 16.86 [1.8-60.44] | 3.90 [1.84-24.39] | | 4.32 | 0.44 | 0.62 | *0.312* |
|  | IL-12 | | 1.7 [0.40-7.96] | 0.91 [0.40-1.31 | | 1.87 | 0.71 | 0.70 | *0.075* |
|  | IL-15 | | 4.10 [4.10-47.22] | 4.10 [4.10-4.10] | | 1,00 | 0.68 | 0.63 | *0.082* |
| Growth factors | | |  |  | |  |  |  |  |
|  | G-CSF | | 102.33 [35.98-1810.32] | 41.33 [29.69-68.25] | | 2.48 | 0.67 | 0.69 | *0.099* |
|  | GM-CSF | | - | - | | - | - | - | *-* |
|  | VEGF | | 0.40 [0.40-86.07] | 0.40 [0.40-0.40] | | 1,00 | 0.97 | 0.71 | *0.020 ^NE^* |
|  | FGF-basic | | 43.60 [8.72-80.69] | 9.86 [6.70-16.75] | | 4.42 | 0.98 | 0.71 | *0.071* |
|  | PDGF-bb | | 18.00 [7.48-80.34] | 9.90 [6.24-15.85] | | 1.82 | 0.54 | 0.69 | *0.089* |
|  |  | | **INFANTS** | | | | | | |
|  |  | | Cytokine concentration  EV meningitis (n=29) | Cytokine concentration control (n=15) | | Fold Change | Effect Size | ROC | *p value* |
| **PLASMA** | | |  |  | |  |  |  |  |
| Chemokines | | |  |  | |  |  |  |  |
|  | MCP-1 | | 146.95 [35.06-313.37] | 36.36 [19.04-83.67] | | 4.04 | 0.85 | 0.73 | *0.014** |
|  | MIP-1a | | 3.39 [2.30-4.34] | 2.87 [2.30-3.90] | | 1.18 | 0,23 | 0.53 | *0.699* |
|  | MIP-1b | | 226.92 [203.05-236.42] | 229.17 [220.95-242.77] | | -1.01 | 0.004 | 0.56 | *0.528* |
|  | Eotaxin | | 28.04 [22.03-35.87] | 34.92 [25.31-49.64] | | -1.25 | -0.47 | 0.63 | *0.166* |
|  | IL-8 | | 13.22 [8.92-25.34] | 14.98 [10.51-23.92] | | -1.13 | -0.21 | 0.54 | *0.647* |
|  | IP-10 | | 3670.8 [1645.98-6487.98] | 879.19 [274.72-2861.01] | | 4.18 | 0.93 | 0.75 | *0.007** |
|  | RANTES | | 5170.96 [4316.74-6580.58] | 4751.41 [4375.80- 7278.03] | | 1.09 | 0.17 | 0.53 | *0.719* |
| Pro-inflammatory response | | | |  | |  |  |  |  |
|  | IL-1β | | 3.38 [2.67-5.07] | 3.3 [2.92-4.61] | | 1.02 | -0.18 | 0.51 | *0.872* |
|  | IL-6 | | 8.59 [2.50-25.38] | 4.49 [3.24-10.09] | | 1.91 | 0.48 | 0.63 | *0.165* |
|  | IFN-γ | | 22.24 [19.20-49.39] | 19.20 [19.20-19.20] | | 1.16 | 0.8 | 0.73 | *0.005* |
|  | TNF-α | | 79.31 [63.63-88.25] | 87.88 [68.64-102.19] | | -1.11 | -0.20 | 0.58 | *0.379* |
|  | IL-17 | | 16.74 [12.94-20.14] | 19.71 [17.02-25.6] | | -1.18 | -0.75 | 0.69 | *0.036** |
| Anti-inflammatory response | | | |  | |  |  |  |  |
|  | IL-1ra | | 1790.42 [699.37-4273.64] | 780.67 [249.34-1470.93] | | 2.29 | 0.78 | 0.72 | *0.016** |
|  | IL-4▪ | | 3.47 [2.46-4.35] | 4.61 [3.44-6.22] | | -1.33 | -0.77 | 0.72 | *0.019** |
|  | IL-10 | | 1.55 [0.80-3.18] | 1.56 [0.80-3.18] | | -1.01 | -0.74 | 0.63 | *0.157* |
|  | IL-13▪ | | 2.00 [2.00-2.12] | 2.22 [2.00-3.14] | | -1.11 | -0.18 | 0.60 | *0.250* |
| Adaptive immunity and lymphocyte activation | | | |  | |  |  |  |  |
|  | IL-2 | | 5.12 [1.63-7.69] | 7.25 [3.36-8.57] | | -1.42 | -0.40 | 0.61 | *0.249* |
|  | IL-5 | | 43.69 [26.48-73.27] | 34.91 [21.68-49.63] | | 1.25 | 0.23 | 0.61 | *0.220* |
|  | IL-7 | | 22.05 [16.23-28.28] | 24.18 [21.24-36.18] | | -1.1 | -0.57 | 0.64 | *0.144* |
|  | IL-9 | | 304.98 [247.08-333.72] | 323.93 [302.13-354.13] | | -1.06 | -0.36 | 0.67 | *0.073* |
|  | IL-12 | | 2.89 [1.72-7.54] | 6.64 [5.03-11.75] | | -2.3 | -.96 | 0.74 | *0.008** |
|  | IL-15 | | 4.10 [4.10-4.10] | 4.10 [4.10-4.10] | | 1.00 | -0.13 | 0.54 | *0.494* |
| Growth factors | | |  |  | |  |  |  |  |
|  | G-CSF | | 168.51 [132.47-216.18] | 148.09 [136.87-210.62] | | 1.14 | 0.02 | 0.54 | *0.701* |
|  | GM-CSF | | 4.40 [4.40-6.19] | 4.40 [4.40-5.08] | | 1.00 | 0.36 | 0.50 | *0.965* |
|  | VEGF | | 0.40 [0.40-0.40] | 0.40 [0.40-0.40] | | 1.00 | 0.01 | 0.50 | *0.985* |
|  | FGF-basic | | 63.97 [52.84-81.41] | 76.53 [64.26-86.88] | | -1.2 | -0.34 | 0.37 | *0.155* |
|  | PDGF-bb | | 608.42 [340.25- 1075.56] | 758.76 [568.83 -939.57] | | -1.25 | -0.29 | 0.60 | *0.270* |
| **CEREBROSPINAL FLUID** | | | |  | |  |  |  |  |
| Chemokines | | |  |  | |  |  |  |  |
|  | MCP-1 | | 1245.04 [740.59 - 1646.55] | 511.785 [428.12-604.35] | | 2.43 | 0.87 | 0.88 | *< 0.001** |
|  | MIP-1a | | 5.61 [2.30-17.74] | 2.30 [2.30-2.30] | | 2.44 | 1.08 | 0.81 | *< 0.001^NE^* |
|  | MIP-1b | | 54.46 [23.07-164.91] | 6.02 [3.69-9.97] | | 9.05 | 1.88 | 0.93 | *< 0.001** |
|  | Eotaxin | | - | - | | - | - | - | *-* |
|  | IL-8 | | 426.59 [149.91-1170.07] | 52.33 [34.96-70.71] | | 8.15 | 1.45 | 0.91 | *< 0.001** |
|  | IP-10 | | 7743.59 [2252.69-13434.49] | 188.03 [108.9-644.34] | | 41.18 | 2.15 | 0.93 | *< 0.001** |
|  | RANTES | | 29.02 [10.81-122.65] | 11.46 [6.35-12.83] | | 2.53 | 0.95 | 0.74 | *0.008* |
| Pro-inflammatory response | | | |  | |  |  |  |  |
|  | IL-1β | | 3.96 [1.73-8.74] | 0.85 [0.70-1.36] | | 4.66 | 1.47 | 0.91 | *< 0.001** |
|  | IL-6 | | 53.06 [1.87-364.71] | 1.00 [1.00-1.00] | | 53.06 | 1.49 | 0.89 | *< 0.001* |
|  | IFN-γ | | 60.71 [19.20-97.40] | 19.20 [19.20-19.20] | | 3.16 | 1.37 | 0.84 | *< 0.001* |
|  | TNF-α | | 20.49 [10.04-38.98] | 2.90 [2.90-2.90] | | 7.07 | 2.10 | 0.97 | *< 0.001** |
|  | IL-17 | | 6.72 [2.94-13.92] | 1.11 [0.66-2.20] | | 6.05 | 1.37 | 0.85 | *< 0.001** |
| Anti-inflammatory response | | | |  | |  |  |  |  |
|  | IL-1ra | | 1020.14 [281.24-4955. 23] | 49.85 [1.30-151.64] | | 20.46 | 1.82 | 0.91 | *< 0.001** |
|  | IL-4▪ | | 1.12 [0.51-2.1] | 0.40 [0.40-0.54] | | 2.8 | 1.27 | 0.86 | *< 0.001** |
|  | IL-10 | | 3.99 [0.81-7.04] | 0.80 [0.80-2.29] | | 4.99 | 0.89 | 0.75 | *0.006** |
|  | IL-13▪ | | 2.00 [2.00-2.00] | 2.00 [2.17-2.37] | | -1.09 | 0.05 | 0.66 | *0.041* |
| Adaptive immunity and lymphocyte activation | | | |  | |  |  |  |  |
|  | IL-2 | | 4.7 [2.04-9.24] | 1.00 [1.00-1.00] | | 4.7 | 1.50 | 0.89 | *< 0.001** |
|  | IL-5 | | 58.78 [27.08-98.07] | 14.42 [9.10-18.75] | | 4.08 | 1.68 | 0.90 | *< 0.001** |
|  | IL-7 | | 6.34 [2.15-9.48] | 1.51 [0.40-2.20] | | 4.20 | 0.99 | 0.76 | *0.005** |
|  | IL-9 | | 21.1 [8.13-88.32] | 3.84 [0.60-5.37] | | 5.49 | 1.70 | 0.93 | *< 0.001** |
|  | IL-12 | | 2.44 [0.90-4.36] | 0.90 [0.40-1.81] | | 2.71 | 0.85 | 0.71 | *0.021** |
|  | IL-15 | | 4.10 [4.10-28,12] | 4.10 [4.10-4.10] | | 1.00 | 0.49 | 0.60 | *0.118* |
| Growth factors | | |  |  | |  |  |  |  |
|  | G-CSF | | 200.71 [99.87-873.09] | 32.14 [20.88-86.04] | | 6.24 | 1.50 | 0.88 | *< 0.001** |
|  | GM-CSF | | - | - | | - | - | - | *-* |
|  | VEGF | | 22.88 [0.40-85.71] | 0.40 [0.40-0.40] | | 57.2 | 1.18 | 0.77 | *0.001** |
|  | FGF-basic | | 44.38 [19.2-67.61] | 6.70 [6.70-8.99] | | 6.62 | 1.87 | 0.90 | *< 0.001** |
|  | PDGF-bb | | 40.02 [16.83-85.05] | 23.30 [10.49-40.67] | | 1.72 | 0.57 | 0.66 | *0.077** |
|  |  | |  |  | |  |  |  |  |
|  |  | |  |  | |  |  |  |  |
|  |  | | CHILDREN | | | | | | |
|  |  | | Cytokine concentration  EV meningitis (n=60) | Cytokine concentration control (n=30) | | Fold Change | Effect Size | ROC | *p value* |
| **PLASMA** | | |  |  | |  |  |  |  |
| Chemokines | | |  |  | |  |  |  |  |
|  | MCP-1 | | 11.05 [8.27-17.95] | 31.21 [18.85-67.94] | | -2.82 | -1.59 | 0.86 | *< 0.001** |
|  | MIP-1a | | 2.30 [2.30-2.30] | 2.30 [2.30-2.92] | | 1,00 | -0.50 | 0.68 | *< 0.001** |
|  | MIP-1b | | 204.44 [187.70-223.03] | 225.16 [194.49-243.28] | | -1.10 | -0.60 | 0.65 | *0.021** |
|  | Eotaxin | | 16.28 [14.50-23.22] | 31.52 [21.09-41.75] | | -1.94 | -1.33 | 0.82 | *< 0.001** |
|  | IL-8 | | 5.22 [3.16-8.40] | 6.37 [4.24-13.86] | | -1.22 | -0.44 | 0.59 | *0.167* |
|  | IP-10 | | 460.21 [302.13-640.54] | 1277.03 [392.72-2621.44] | | -2.77 | -0.93 | 0.71 | *0.001** |
|  | RANTES | | 5027.81 [3856.10-6170.10] | 6936.32 [4470.84-8839.52] | | -1.38 | -0.51 | 0.65 | *0.021** |
| Pro-inflammatory response | | | |  | |  |  |  |  |
|  | IL-1β | | 2.23 [1.82-2.83] | 3.10 [2.55-4.93] | | -1.39 | -0.69 | 0.78 | *< 0.001* |
|  | IL-6 | | 6.48 [3.37-15.22] | 9.67 [2.58-21.58] | | -1.49 | -0.24 | 0.56 | *0.331* |
|  | IFN-γ | | 19.20 [19.20-19.20] | 19.20 [19.20-24.03] | | 1.00 | -0.79 | 0.63 | *< 0,001** |
|  | TNF-α | | 51.75 [46.01-59.65] | 73.22 [61.05-91.60] | | -1.41 | -1.19 | 0.82 | *< 0,001** |
|  | IL-17 | | 12.38 [10.76-14.26] | 15.21 [13.26-17.89] | | -1.23 | -0.73 | 0.73 | *< 0,001** |
| Anti-inflammatory response | | | |  | |  |  |  |  |
|  | IL-1ra | | 466.94 [195.80-873.00] | 1217.32 [597.55-3204.93] | | -2.61 | -0.81 | 0.77 | *< 0.001** |
|  | IL-4▪ | | 2.43 [2.02-3.17] | 4.23 [3.29-5.04] | | -1.74 | -1.18 | 0.80 | *< 0.001** |
|  | IL-10 | | 0.80 [0.80-1.08] | 5.08 [1.32-15.29] | | -6.35 | -1.63 | 0.83 | *< 0.001** |
|  | IL-13▪ | | 2.00 [2.00-2.44] | 2.15 [2.00-3.01] | | -1.075 | -0.59 | 0.62 | *0.034* |
| Adaptive immunity and lymphocyte activation | | | |  | |  |  |  |  |
|  | IL-2 | | 2.04 [1.00-3.95] | 3.83 [2.28-8.13] | | -1.88 | -0.76 | 0.68 | *0.004** |
|  | IL-5 | | 22.34 [8.71-42.04] | 35.80 [13.21-67.29] | | -1.60 | -0.34 | 0.62 | *0.066* |
|  | IL-7 | | 17.23 [12.78-22.47] | 21.59 [19.10-29.34] | | -1.25 | -0.56 | 0.69 | *0.003* |
|  | IL-9 | | 285.62 [246.26-322.88] | 312.55 [269.55-347.46] | | -1.09 | -0.60 | 0.65 | *0.023** |
|  | IL-12 | | 3.70 [1.61-8.34] | 4.89 [4.18-13.43] | | -1.32 | -0.63 | 0.66 | *0.015* |
|  | IL-15 | | 4.10 [4.10-4.10] | 4.10 [4.10-200.74] | | 1.00 | -0.53 | 0.59 | *0.021** |
| Growth factors | | |  |  | |  |  |  |  |
|  | G-CSF | | 82.17 [63.76-105.17] | 125.16 [103.37-181.47] | | -1.52 | -0.99 | 0.78 | *< 0.001** |
|  | GM-CSF | | 4.40 [4.40-5.23] | 4.40 [4.40-6.40] | | 1.00 | -0.21 | 0.53 | *0.530* |
|  | VEGF | | 0.40 [0.40-0.40] | 0.40 [0.40-201.57] | | 1.00 | -0.68 | 0.63 | *0.003** |
|  | FGF-basic | | 57.38 [46.59-66.17] | 64.97 [54.75-86.99] | | -1.13 | -0.63 | 0.64 | *0.033** |
|  | PDGF-bb | | 692.68 [413.65-1192.36] | 724.76 [371.72-1180.90] | | -1.05 | 0.02 | 0.51 | *0.871* |
| **CEREBROSPINAL FLUID** | | | |  | |  |  |  |  |
| Chemokines | | |  |  | |  |  |  |  |
|  | MCP-1 | | 1309.94 [674.11-2085.58] | 352.79 [153.05-1055.16] | | 3.71 | 0.95 | 0.77 | *< 0.001** |
|  | MIP-1a | | 5.59 [3.67-9.26] | 2.30 [2.30-2.30] | | 2.43 | 1.49 | 0.92 | *< 0.001* |
|  | MIP-1b | | 48.77 [34.87-103.63] | 6.73 [3.98-9.85] | | 7.25 | 2.64 | 0.98 | *< 0.001** |
|  | Eotaxin | | - | - | | - | - | - | *-* |
|  | IL-8 | | 1946.39 [1013.15-5576.32] | 75.16 [37.14-105.69] | | 25.90 | 2.85 | 0.98 | *< 0.001** |
|  | IP-10 | 18701.94 [14508.30-26808.50] | | | 358.26 [112.18-1594.00] | 52.20 | 2.71 | 0.98 | *< 0.001** |
|  | RANTES | | 26.80 [21.12-43.32] | 11.77 [8.25-13.95] | | 2.28 | 1.17 | 0.92 | *< 0.001** |
| Pro-inflammatory response | | | |  | |  |  |  |  |
|  | IL-1β | | 7.40 [5.96-15.86] | 0.70 [0.70-0.98] | | 10.56 | 2.82 | 0.98 | *< 0.001** |
|  | IL-6 | | 990.74 [446.73-2251.23] | 5.91 [2.42-13.81] | | 167.71 | 3.58 | 0.98 | *< 0.001** |
|  | IFN-γ | | 77.02 [47.95-151.24] | 19.20 [19.20-21.36] | | 4.01 | 1.94 | 0.93 | *< 0.001** |
|  | TNF-α | | 34.93 [23.55-49.72] | 2.90 [2.90-4.26] | | 12.04 | 3.38 | 0.98 | *< 0.001** |
|  | IL-17 | | 13.25 [9.08-18.43] | 1.08 [0.66-1.70] | | 12.32 | 3.32 | 0.98 | *< 0.001** |
| Anti-inflammatory response | | | |  | |  |  |  |  |
|  | IL-1ra | | 6640.44 [3487.17-8690.14] | 96.60 [54.60-179.75] | | 68.74 | 3.39 | 0.98 | *< 0.001** |
|  | IL-4▪ | | 2.44 [1.70-3.69] | 0.44 [0.40-0.52] | | 5.54 | 2.26 | 0.98 | *< 0.001** |
|  | IL-10 | | 28.23 [14.61-44.43] | 3.06 [2.26-3.75] | | 9.24 | 2.75 | 0.98 | *< 0.001** |
|  | IL-13▪ | | 2.00 [2.00-2.06] | 2.00 [2.00-2.18] | | 1,00 | 0.10 | 0.49 | *0.867* |
| Adaptive immunity and lymphocyte activation | | | |  | |  |  |  |  |
|  | IL-2 | | 9.44 [7.06-12.70] | 1.16 [1.00-1.73] | | 8.14 | 3.28 | 0.98 | *< 0.001** |
|  | IL-5 | | 161.84 [103.98-203.34] | 30.11 [20.77-35.21] | | 5.37 | 2.99 | 0.99 | *< 0.001** |
|  | IL-7 | | 7.67 [4.34-14.30] | 0.40 [0.40-0.40] | | 19.18 | 2.05 | 0.93 | *< 0.001** |
|  | IL-9 | | 50.22 [33.06-75.09] | 7.54 [4.74-9.08] | | 6.66 | 3.06 | 0.99 | *< 0.001** |
|  | IL-12 | | 5.53 [4.10-8.83] | 1.53 [0.40-2.02] | | 3.63 | 1.74 | 0.91 | *< 0.001** |
|  | IL-15 | | 115.27 [4.10-186.36] | 56.85 [4.10-121.16] | | 2.03 | 0.10 | 0.60 | *0.136* |
| Growth factors | | |  |  | |  |  |  |  |
|  | G-CSF | | 383.74 [185.33-730.70] | 49.53 [35.57-92.12] | | 7.75 | 1.98 | 0.92 | *< 0.001** |
|  | GM-CSF | | - | - | | - | - | - | *-* |
|  | VEGF | | 131.69 [91.79-150.55] | 61.96 [39.14-99.60] | | 2.13 | 1.12 | 0.83 | *< 0.001** |
|  | FGF-basic | | 59.52 [50.16-76.03] | 8.67 [6.70-12.13] | | 6.86 | 3.93 | 0.98 | *< 0.001** |
|  | PDGF-bb | | 70.28 [52.92-104.66] | 25.94 [18.92-38.45] | | 2.67 | 1.59 | 0.88 | *< 0.001** |

Cytokine-chemokine expression (pg/mL) is expressed in median [IQR] for EV (EV meningitis) and control groups. Fold change, effect size, receiver operating characteristic (ROC) and p value between EV and control group are indicated for each cytokine/chemokine. For comparisons of EV meningitis and control patients, a multivariate analysis adjusted for sample collection time was performed; p values that remained significant after multivariate analysis adjustment are indicated with *, and those not evaluated with ^NE^.

**Table S4.** Expression of cytokines and chemokines in neonates, infants and children with EV meningitis (restricted to PCR-positive results for EV in the CSF) compared with control patients.

|  |  | | **NEONATES** | | | | | | |
| --- | --- | --- | --- | --- | --- | --- | --- | --- | --- |
|  |  | | Cytokine concentration  CSF EV+ (n=17) | Cytokine concentration Control (n=10) | | Fold Change | Effect Size | ROC | *p value* |
| **PLASMA** | | |  |  | |  |  |  |  |
| Chemokines | | |  |  | |  |  |  |  |
|  | MCP-1 | | 487.96 [83.85–701.94] | 46.92 [29.99-54.74] | | 10.40 | 1.16 | 0.83 | *0.005* |
|  | MIP-1a | | 5.44 [3.73–6.12] | 2.70 [2.30-3.93] | | 2.01 | 1.33 | 0.86 | *0.002* |
|  | MIP-1b | | 221.87 [214.20–256.04] | 220.20 [206.13-241.85] | | 1.01 | 0.16 | 0.57 | *0.581* |
|  | Eotaxin | | 36.12 [26.91–43.66] | 41.61 [34.86-51.14] | | -1.15 | -0.32 | 0.61 | *0.367* |
|  | IL-8 | | 23.76 [19.13–28.08] | 14.39 [8.06-19.05] | | 1.65 | 0.94 | 0.82 | *0.007* |
|  | IP-10 | | 6148.58 [3792.40–7564.10] | 610.70 [255.07-3543.98] | | 10.07 | 1.42 | 0.80 | *0.01* |
|  | RANTES | | 4516.00 [4362.60–6333.31] | 5348.81 [4423.32-7643.51] | | -1.18 | -0.02 | 0.46 | *0.725* |
| Pro-inflammatory response | | | |  | |  |  |  |  |
|  | IL-1β | | 4.70 [3.72–5.85] | 3.07 [2.67-4.24] | | 1.53 | 0.76 | 0.73 | *0.05* |
|  | IL-6 | | 22.78 [12.71–53.62] | 3.92 [2.74-8.06] | | 5.81 | 1.62 | 0.92 | *<0.001* |
|  | IFN-γ | | 73.71 [26.18–113.37] | 19.20 [19.20-20.45] | | 3.84 | 1.35 | 0.84 | *0.003* |
|  | TNF-α | | 88.38 [75.77–94.97] | 83.22 [64.08-98.60] | | 1.06 | 0.25 | 0.57 | *0.581* |
|  | IL-17 | | 20.29 [17.78–23.13] | 17.64 [16.33-20.48] | | 1.15 | 0.12 | 0.58 | *0.498* |
| Anti-inflammatory response | | | |  | |  |  |  |  |
|  | IL-1ra | | 4605.04 [3417.92–6322.31] | 473.31 [114.05-2002.55] | | 9.73 | 1.53 | 0.89 | *0.001* |
|  | IL-4 | | 4.62 [3.19–5.23] | 4.55 [4.08-5.05] | | 1.02 | -0.04 | 0.51 | *0.94* |
|  | IL-10 | | 1.48 [0.8–3.89] | 3.09 [0.80-7.15] | | -2.09 | -0.35 | 0.60 | *0.371* |
|  | IL-13 | | 2.00 [2.00-2.49] | 2.12 [2.00-2.40] | | -1.06 | 0.03 | 0.53 | *0.77* |
| Adaptive immunity and lymphocyte activation | | | |  | |  |  |  |  |
|  | IL-2 | | 8.76 [6.77–10.71] | 4.32 [2.73-6.77] | | 2.03 | 1.42 | 0.85 | *0.003* |
|  | IL-5 | | 93.19 [53.59–115.96] | 40.62 [14.38-58.32] | | 2.29 | 1.11 | 0.79 | *0.014* |
|  | IL-7 | | 27.95 [22.52–30.73] | 27.94 [24.36-35.05] | | 1.00 | -0.45 | 0.59 | *0.422* |
|  | IL-9 | | 296.29 [246.11–352.61] | 310.68 [277.42-346.29] | | 0.95 | -0.23 | 0.56 | *0.616* |
|  | IL-12 | | 8.29 [2.31–9.63] | 4.17 [1.31-6.09] | | 1.99 | 0.62 | 0.71 | *0.079* |
|  | IL-15 | | 4.10 [4.10–4.10] | 4.10 [4.10-4.10] | | 1.00 | 0.26 | 0.54 | *0.679* |
| Growth factors | | |  |  | |  |  |  |  |
|  | G-CSF | | 252.71 [188.44–296.57] | 148.74 [134.28-239.51] | | 1.70 | 0.86 | 0.75 | *0.035* |
|  | GM-CSF | | 5.78 [4.40–7.34] | 4.40 [4.40-4.87] | | 1.31 | 0.80 | 0.73 | *0.043* |
|  | VEGF | | 0.40 [0.40–63.01] | 0.40 [0.40-3.22] | | 1.00 | 0.19 | 0.52 | *0.834* |
|  | FGF-basic | | 80.10 [69.57–90.74] | 58.40 [53.51-64.96] | | 1.37 | 1.41 | 0.87 | *0.001* |
|  | PDGF-bb | | 501.32 [301.86–809.83] | 445.51 [307.12-1070.37] | | 1.13 | 0.22 | 0.51 | *0.92* |
| **CEREBROSPINAL FLUID** | | | |  | |  |  |  |  |
| Chemokines | | |  |  | |  |  |  |  |
|  | MCP-1 | | 1195.18 [864.99–1572.60] | 722.41 [571.47-847.90] | | 1.65 | 1.14 | 0.82 | *0.006* |
|  | MIP-1a | | 6.14 [2.30–39.93] | 2.30 [2.30-2.30] | | 2.67 | 0.93 | 0.72 | *0.037* |
|  | MIP-1b | | 59.71 [15.78–195.96] | 11.17 [6.36-17.89] | | 5.35 | 1.24 | 0.82 | *0.006* |
|  | Eotaxin | | - | - | | - | - | - | *-* |
|  | IL-8 | | 590.43 [123.36–3205.56] | 66.37 [53.95-109.32] | | 8.90 | 1.52 | 0.92 | *<0.001* |
|  | IP-10 | | 10050.35 [2143.50–15865.82] | 688.90 [189.34-1177.58] | | 14.59 | 1.73 | 0.88 | *0.001* |
|  | RANTES | | 20.62 [5.81–79.89] | 6.72 [6.09-73.11] | | 3.07 | 0.23 | 0.56 | *0.633* |
| Pro-inflammatory response | | | |  | |  |  |  |  |
|  | IL-1β | | 4.84 [1.14–19.75] | 1.13 [1.01-1.22] | | 4.28 | 1.13 | 0.77 | *0.021* |
|  | IL-6 | | 13.19 [1.31–1786.02] | 1.00 [1.00-1.00] | | 13.19 | 1.26 | 0.90 | *<0.001* |
|  | IFN-γ | | 75.19 [19.81–129.12] | 19.20 [19.20-20.12] | | 3.92 | 1.31 | 0.81 | *0.006* |
|  | TNF-α | | 17.73 [9.53–73.32] | 6.77 [3.05-8.94] | | 2.62 | 1.18 | 0.82 | *0.007* |
|  | IL-17 | | 8.10 [1.29–19.61] | 1.58 [1.00-1.95] | | 5.13 | 0.92 | 0.70 | *0.082* |
| Anti-inflammatory response | | | |  | |  |  |  |  |
|  | IL-1ra | | 1069.85 [302.87–9849.79] | 110.94 [1.30-156.98] | | 9.64 | 1.86 | 0.97 | *<0.001* |
|  | IL-4▪ | | 1.08 [0.44–3.28] | 0.40 [0.40-0.40] | | 2.70 | 1.15 | 0.85 | *0.002* |
|  | IL-10 | | 0.80 [0.80–10.21] | 0.80 [0.80-0.80] | | 1.00 | 0.83 | 0.65 | *0.135* |
|  | IL-13▪ | | 2.00 [2.00-2.00] | 2.00 [2.00-2.00] | | 1.00 | -0.34 | 0.48 | *0.745* |
| Adaptive immunity and lymphocyte activation | | | |  | |  |  |  |  |
|  | IL-2 | | 6.37 [1.00–17.31] | 1.00 [1.00-1.52] | | 6.37 | 1.10 | 0.71 | *0.055* |
|  | IL-5 | | 66.04 [14.41–185.23] | 13.12 [3.74-20.77] | | 5.03 | 1.43 | 0.81 | *0.007* |
|  | IL-7 | | 7.31 [5.83–9.35] | 5.38 [1.51-6.03] | | 1.36 | 0.59 | 0.69 | *0.102* |
|  | IL-9 | | 34.64 [3.49–60.44] | 3.90 [1.84-24.39] | | 8.88 | 0.64 | 0.68 | *0.119* |
|  | IL-12 | | 1.97 [1.39–7.96] | 0.91 [0.40-1.31 | | 2.16 | 0.86 | 0.76 | *0.025* |
|  | IL-15 | | 4.10 [4.10–47.22] | 4.10 [4.10-4.10] | | 1.00 | 0.74 | 0.65 | *0.064* |
| Growth factors | | |  |  | |  |  |  |  |
|  | G-CSF | | 164.11 [42.495–1810.32] | 41.33 [29.69-68.25] | | 3.97 | 0.98 | 0.75 | *0.035* |
|  | GM-CSF | | - | - | | - | - | - | *-* |
|  | VEGF | | 0.40 [0.40–86.07] | 0.40 [0.40-0.40] | | 1.00 | 1.09 | 0.74 | *0.045* |
|  | FGF-basic | | 54.32 [9.50–80.69] | 9.86 [6.70-16.75] | | 5.51 | 1.20 | 0.77 | *0.023* |
|  | PDGF-bb | | 20.71 [13.72–80.34] | 9.90 [6.24-15.85] | | 2.09 | 0.85 | 0.77 | *0.021* |
|  |  | |  |  | |  |  |  |  |
|  |  | | **INFANTS** | | | | | | |
|  |  | | Cytokine concentration  CSF EV+ (n=22) | Cytokine concentration control (n=15) | | Fold Change | Effect Size | ROC | *p value* |
| **PLASMA** | | |  |  | |  |  |  |  |
| Chemokines | | |  |  | |  |  |  |  |
|  | MCP-1 | | 167.17 [26.25–431.80] | 36.36 [19.04–83.67] | | 4.60 | 0.81 | 0.70 | *0.041* |
|  | MIP-1a | | 3.33 [2.30–4.54] | 2.87 [2.30–3.90] | | 1.16 | 0.27 | 0.54 | *0.682* |
|  | MIP-1b | | 225.22 [200.75–249.04] | 229.17 [220.95–242.77] | | 0.98 | 0.03 | 0.55 | *0.621* |
|  | Eotaxin | | 26.80 [20.23–32.17] | 34.92 [25.31–49.64] | | -1.30 | -0.67 | 0.68 | *0.07* |
|  | IL-8 | | 14.13 [8.57–26.23] | 14.98 [10.51–23.92] | | -1.06 | -0.13 | 0.52 | *0.877* |
|  | IP-10 | | 4636.06 [1645.98–8055.86] | 879.19 [274.72–2861.01] | | 5.27 | 0.90 | 0.76 | *0.009* |
|  | RANTES | | 5039.95 [4310.00–6577.69] | 4751.41 [4375.80–7278.03] | | 1.06 | 0.16 | 0.52 | *0.877* |
| Pro-inflammatory response | | | |  | |  |  |  |  |
|  | IL-1β | | 3.19 [2.55–5.07] | 3.30 [2.92–4.61] | | 0.97 | -0.28 | 0.54 | *0.676* |
|  | IL-6 | | 8.14 [2.30–25.38] | 4.49 [3.24–10.09] | | 1.81 | 0.38 | 0.58 | *0.439* |
|  | IFN-γ | | 23.13 [19.20–63.36] | 19.20 [19.20–19.20] | | 1.20 | 0.95 | 0.75 | *0.005* |
|  | TNF-α | | 78.80 [63.63–86.74] | 87.88 [68.64–102.19] | | -1.12 | -0.20 | 0.61 | *0.279* |
|  | IL-17 | | 16.39 [12.18–18.94] | 19.71 [17.02–25.60] | | -1.20 | -0.99 | 0.76 | *0.007* |
| Anti-inflammatory response | | | |  | |  |  |  |  |
|  | IL-1ra | | 1999.19 [591.80–4682.07] | 780.67 [249.34–1470.93] | | 2.56 | 0.70 | 0.71 | *0.03* |
|  | IL-4▪ | | 3.20 [2.31–3.72] | 4.61 [3.44–6.22] | | -1.44 | -1.06 | 0.78 | *0.004* |
|  | IL-10 | | 1.20 [0.80–2.96] | 4.47 [0.80–14.31] | | -3.73 | -0.76 | 0.65 | *0.112* |
|  | IL-13▪ | | 2.00 [2.00–2.04] | 2.22 [2.00–3.14] | | -1.11 | -0.34 | 0.62 | *0.173* |
| Adaptive immunity and lymphocyte activation | | | |  | |  |  |  |  |
|  | IL-2 | | 4.11 [1.63–7.71] | 7.25 [3.36–8.57] | | -1.76 | -0.42 | 0.61 | *0.265* |
|  | IL-5 | | 44.75 [14.34–76.07] | 34.91 [21.68–49.63] | | 1.28 | 0.17 | 0.61 | *0.265* |
|  | IL-7 | | 20.65 [16.23–27.36] | 24.18 [21.24–36.18] | | -1.17 | -0.73 | 0.67 | *0.075* |
|  | IL-9 | | 276.80 [237.13–315.43] | 323.92 [302.13–354.13] | | -1.17 | -0.47 | 0.74 | *0.016* |
|  | IL-12 | | 2.72 [1.12–7.54] | 6.64 [5.03–11.75] | | -2.44 | -1.07 | 0.76 | *0.009* |
|  | IL-15 | | 4.10 [4.10–4.10] | 4.10 [4.10–4.10] | | 1.00 | -0.20 | 0.55 | *0.435* |
| Growth factors | | |  |  | |  |  |  |  |
|  | G-CSF | | 168.88 [111.64–218.20] | 148.09 [136.87–210.62] | | 1.14 | -0.04 | 0.49 | *0.902* |
|  | GM-CSF | | 4.40 [4.40–6.19] | 4.40 [4.40–5.08] | | 1.00 | 0.34 | 0.52 | *0.853* |
|  | VEGF | | 0.40 [0.40–0.40] | 0.40 [0.40–0.40] | | 1.00 | -0.08 | 0.52 | *0.736* |
|  | FGF-basic | | 61.00 [49.54–80.30] | 76.53 [64.26–86.88] | | -1.25 | -0.44 | 0.67 | *0.078* |
|  | PDGF-bb | | 622.67 [310.07–1075.56] | 758.76 [568.83–939.57] | | -1.22 | -0.34 | 0.60 | *0.307* |
| **CEREBROSPINAL FLUID** | | | |  | |  |  |  |  |
| Chemokines | | |  |  | |  |  |  |  |
|  | MCP-1 | | 1328.85 [718.84–1741.81] | 511.79 [428.12–604.35] | | 2.60 | 0.84 | 0.87 | *<0.001* |
|  | MIP-1a | | 5.67 [2.30–23.04] | 2.30 [2.30–2.30] | | 2.47 | 1.22 | 0.84 | *<0.001* |
|  | MIP-1b | | 97.80 [23.69–176.19] | 6.02 [3.69–9.97] | | 16.25 | 2.25 | 0.96 | *<0.001* |
|  | Eotaxin | | - | - | | - | - | - | *-* |
|  | IL-8 | | 457.42 [159.45–1246.86] | 52.33 [34.96–70.61] | | 8.74 | 1.53 | 0.92 | *<0.001* |
|  | IP-10 | | 11113.96 [2407.88–13813.13] | 188.03 [108.90–644.34] | | 59.11 | 2.88 | 0.97 | *<0.001* |
|  | RANTES | | 41.478 [8.67–343.72] | 11.46 [6.35–12.83] | | 3.62 | 1.04 | 0.73 | *0.017* |
| Pro-inflammatory response | | | |  | |  |  |  |  |
|  | IL-1β | | 4.62 [2.45–10.70] | 0.85 [7.00–1.36] | | 5.44 | 1.84 | 0.97 | *<0.001* |
|  | IL-6 | | 74.69 [1.53–492.55] | 1.00 [1.00–1.00] | | 74.69 | 1.63 | 0.88 | *<0.001* |
|  | IFN-γ | | 79.16 [38.15–150.94] | 19.20 [19.20–19.20] | | 4.12 | 1.85 | 0.89 | *<0.001* |
|  | TNF-α | | 29.89 [12.75–62.88] | 2.90 [2.90–2.90] | | 10.31 | 2.62 | 0.99 | *<0.001* |
|  | IL-17 | | 7.06 [3.10–14.6] | 1.11 [0.66–2.20] | | 6.36 | 1.77 | 0.91 | *<0.001* |
| Anti-inflammatory response | | | |  | |  |  |  |  |
|  | IL-1ra | | 1147.45 [338.62–9110.90] | 49.85 [1.30–151.64] | | 23.02 | 2.19 | 0.94 | *<0.001* |
|  | IL-4▪ | | 1.23 [0.51–2.41] | 0.4.00 [0.4.00–0.54] | | 3.08 | 1.44 | 0.87 | *<0.001* |
|  | IL-10 | | 3.513 [0.81–8.86] | 0.80 [0.80–2.29] | | 4.39 | 0.95 | 0.75 | *0.009* |
|  | IL-13▪ | | 2.00 [2.00–2.00] | 2.17 [2.00–2.37] | | -1.09 | 0.10 | 0.37 | *0.141* |
| Adaptive immunity and lymphocyte activation | | | |  | |  |  |  |  |
|  | IL-2 | | 5.06 [2.92–11.26] | 1.00 [1.00–1.00] | | 5.06 | 1.92 | 0.93 | *<0.001* |
|  | IL-5 | | 66.04 [27.27–116.80] | 14.42 [9.10–18.75] | | 4.58 | 1.74 | 0.90 | *<0.001* |
|  | IL-7 | | 7.02 [2.58–10.51] | 1.51 [0.40–2.20] | | 4.65 | 1.17 | 0.80 | *<0.001* |
|  | IL-9 | | 25.38 [11.12–99.89] | 3.84 [0.60–5.37] | | 6.61 | 1.98 | 0.95 | *<0.001* |
|  | IL-12 | | 2.89 [0.90–6.30] | 0.90 [0.40–1.81] | | 3.21 | 1.04 | 0.75 | *0.001* |
|  | IL-15 | | 4.10 [4.10–28.12] | 4.10 [4.10–4.10] | | 1.00 | 0.49 | 0.60 | *0.135* |
| Growth factors | | |  |  | |  |  |  |  |
|  | G-CSF | | 189.29 [99.87–1060.44] | 32.14 [20.88–86.04] | | 5.89 | 1.60 | 0.89 | *<0.001* |
|  | GM-CSF | | - | - | | - | - | - | *-* |
|  | VEGF | | 25.934 [0.4–85.71] | 0.40 [0.40–0.40] | | 64.84 | 1.25 | 0.78 | *0.001* |
|  | FGF-basic | | 55.16 [27.74–72.01] | 6.70 [6.70–9.00] | | 8.23 | 2.45 | 0.93 | *<0.001* |
|  | PDGF-bb | | 47.10 [12.97–112.20] | 23.30 [10.49–40.67] | | 2.02 | 0.55 | 0.64 | *0.146* |
|  |  | |  |  | |  |  |  |  |
|  |  | |  |  | |  |  |  |  |
|  |  | |  |  | |  |  |  |  |
|  |  | | **CHILDREN** | | | | | | |
|  |  | | Cytokine concentration  CSF EV+ (n=57) | Cytokine concentration control (n=30) | | Fold Change | Effect Size | ROC | *p value* |
| **PLASMA** | | |  |  | |  |  |  |  |
| Chemokines | | |  |  | |  |  |  |  |
|  | MCP-1 | | 10.85 [8.22–16.03] | 31.21 [18.85–67.94] | | -2.88 | -1.70 | 0.88 | *<0.001* |
|  | MIP-1a | | 2.30 [2.30–2.30] | 2.30 [2.30–2.92] | | 1.00 | -0.49 | 0.68 | *<0.001* |
|  | MIP-1b | | 204.58 [189.22–223.14] | 225.16 [194.49–243.28] | | -1.10 | -0.56 | 0.64 | *0.035* |
|  | Eotaxin | | 15.88 [14.50-22.156] | 31.52 [21.09-41.75] | | -1.98 | -1.50 | 0.84 | *<0.001* |
|  | IL-8 | | 5.18 [3.16–8.26] | 6.37 [4.24–13.86] | | -1.23 | -0.47 | 0.60 | *0.118* |
|  | IP-10 | | 427.87 [301.03–595.98] | 1277.03 [392.72–2621.44] | | -2.98 | -1.00 | 0.72 | *<0.001* |
|  | RANTES | | 5031.84 [3879.30–6198.69] | 6936.32 [4470.84–8839.52] | | -1.38 | -0.49 | 0.65 | *0.024* |
| Pro-inflammatory response | | | |  | |  |  |  |  |
|  | IL-1β | | 2.17 [1.81–2.75] | 3.10 [2.55–4.93] | | -1.43 | -0.69 | 0.79 | *<0.001* |
|  | IL-6 | | 6.46 [3.36–15.43] | 9.67 [2.58–21.58] | | -1.50 | -0.24 | 0.56 | *0.346* |
|  | IFN-γ | | 19.20 [19.20–19.20] | 19.20 [19.20–24.03] | | 1.00 | -0.77 | 0.65 | *<0.001* |
|  | TNF-α | | 51.50 [46.27–60.44] | 73.22 [61.05–91.60] | | -1.42 | -1.16 | 0.81 | *<0.001* |
|  | IL-17 | | 12.37 [10.66–14.25] | 15.21 [13.26–17.89] | | -1.23 | -0.75 | 0.74 | *<0.001* |
| Anti-inflammatory response | | | |  | |  |  |  |  |
|  | IL-1ra | | 433.90 [191.11–715.10] | 1217.32 [597.55–3204.93] | | -2.81 | -0.85 | 0.78 | *<0.001* |
|  | IL-4▪ | | 2.37 [2.02–3.12] | 4.23 [3.29–5.04] | | -1.78 | -1.31 | 0.83 | *<0.001* |
|  | IL-10 | | 0.80 [0.80–1.08] | 5.08 [1.32–15.29] | | -6.35 | -1.59 | 0.83 | *<0.001* |
|  | IL-13▪ | | 2.00 [2.00–2.44] | 2.15 [2.00–3.01] | | -1.08 | -0.56 | 0.61 | *0.052* |
| Adaptive immunity and lymphocyte activation | | | |  | |  |  |  |  |
|  | IL-2 | | 1.88 [1.00–3.95] | 3.83 [2.28–8.13] | | -2.04 | -0.77 | 0.69 | *0.003* |
|  | IL-5 | | 23.01 [7.97–42.78] | 35.80 [13.21–67.29] | | -1.56 | -0.34 | 0.61 | *0.083* |
|  | IL-7 | | 17.11 [12.64–22.18] | 21.59 [19.10–29.34] | | -1.26 | -0.58 | 0.70 | *0.002* |
|  | IL-9 | | 286.53 [250.76–322.92] | 312.55 [269.55–347.46] | | -1.09 | -0.57 | 0.64 | *0.037* |
|  | IL-12 | | 3.57 [1.50–8.29] | 4.89 [4.18–13.43] | | -1.37 | -0.66 | 0.67 | *0.008* |
|  | IL-15 | | 4.10 [4.10–4.10] | 4.10 [4.10–200.74] | | 1.00 | -0.51 | 0.59 | *0.028* |
| Growth factors | | |  |  | |  |  |  |  |
|  | G-CSF | | 81.85 [62.77–100.90] | 125.16 [103.37–181.47] | | -1.53 | -1.03 | 0.79 | *<0.001* |
|  | GM-CSF | | 4.40 [4.40–5.27] | 4.40 [4.40–6.40] | | 1.00 | -0.19 | 0.53 | *0.555* |
|  | VEGF | | 0.40 [0.40–0.40] | 0.4 [0.4–201.57] | | 1.00 | -0.65 | 0.63 | *0.004* |
|  | FGF-basic | | 56.49 [46.59–65.75] | 64.97 [54.75–86.99] | | -1.15 | -0.66 | 0.65 | *0.022* |
|  | PDGF-bb | | 684.09 [407.26–1153.85] | 724.76 [371.72–1180.90] | | -1.06 | -0.0002 | 0.50 | *0.957* |
| **CEREBROSPINAL FLUID** | | | |  | |  |  |  |  |
| Chemokines | | |  |  | |  |  |  |  |
|  | MCP-1 | | 1315.41 [729.06–2118.71] | 352.78 [153.05–1055.16] | | 3.73 | 1.03 | 0.78 | *<0.001* |
|  | MIP-1a | | 5.78 [3.79–9.44] | 2.30 [2.30–2.30] | | 2.51 | 1.61 | 0.94 | *<0.001* |
|  | MIP-1b | | 51.05 [36.59–104.45] | 6.73 [3.98–9.85] | | 7.59 | 2.95 | 1.00 | *<0.001* |
|  | Eotaxin | | - | - | | - | - | - | *-* |
|  | IL-8 | | 1999.13 [1016.39–5605.37] | 75.16 [37.14–105.69] | | 26.61 | 3.22 | 0.99 | *<0.001* |
|  | IP-10 | 19218.03 [14839.28–27122.46] | | | 358.26 [112.18–1594.00] | 53.64 | 3.65 | 1.00 | *<0.001* |
|  | RANTES | | 27.11 [21.33–46.20] | 11.77 [8.25–13.95] | | 2.30 | 1.22 | 0.94 | *<0.001* |
| Pro-inflammatory response | | | |  | |  |  |  |  |
|  | IL-1β | | 7.43 [6.25–16.32] | 0.70 [0.70-0.98] | | 10.61 | 3.28 | 1.00 | *<0.001* |
|  | IL-6 | | 1069.71 [618.25–2269.00] | 5.91 [2.42–13.81] | | 181.00 | 4.23 | 0.99 | *<0.001* |
|  | IFN-γ | | 77.71 [53.64–151.27] | 19.20 [19.20-21.36] | | 4.05 | 2.15 | 1.00 | *<0.001* |
|  | TNF-α | | 36.15 [25.97–49.72] | 2.90 [2.90–4.26] | | 12.47 | 4.46 | 1.00 | *<0.001* |
|  | IL-17 | | 13.36 [9.32–18.49] | 1.08 [0.66–1.70] | | 12.37 | 4.02 | 1.00 | *<0.001* |
| Anti-inflammatory response | | | |  | |  |  |  |  |
|  | IL-1ra | | 6711.20 [3770.59–8844.99] | 96.60 [54.6-0179.75] | | 69.47 | 4.52 | 1.00 | *<0.001* |
|  | IL-4▪ | | 2.49 [1.87–3.81] | 0.44 [0.40–0.52] | | 5.66 | 2.52 | 0.99 | *<0.001* |
|  | IL-10 | | 30.35 [16.11–46.98] | 3.06 [2.26–3.75] | | 9.92 | 3.21 | 1.00 | *<0.001* |
|  | IL-13▪ | | 2.00 [2.00–2.00] | 2.00 [2.00–2.18] | | 1.00 | 0.07 | 0.52 | *0.707* |
| Adaptive immunity and lymphocyte activation | | | |  | |  |  |  |  |
|  | IL-2 | | 9.68 [7.29–12.73] | 1.16 [1.00-1.73] | | 8.34 | 3.95 | 1.00 | *<0.001* |
|  | IL-5 | | 168.30 [111.97–205.02] | 30.11 [20.77-35.21] | | 5.59 | 3.24 | 1.00 | *<0.001* |
|  | IL-7 | | 7.95 [4.94–14.72] | 0.4 [0.4–0.4] | | 19.88 | 2.35 | 0.96 | *<0.001* |
|  | IL-9 | | 52.67 [35.09–76.97] | 7.54 [4.74–9.08] | | 6.99 | 3.33 | 1.00 | *<0.001* |
|  | IL-12 | | 5.90 [4.10–8.97] | 1.53 [0.40–2.02] | | 3.86 | 1.87 | 0.93 | *<0.001* |
|  | IL-15 | | 121.51 [4.10–180.50] | 56.85 [4.10–121.16] | | 2.14 | 0.19 | 0.59 | *0.147* |
| Growth factors | | |  |  | |  |  |  |  |
|  | G-CSF | | 421.37 [210.49–757.75] | 49.53 [35.57–92.12] | | 8.51 | 2.10 | 0.94 | *<0.001* |
|  | GM-CSF | | - | - | | - | - | - | *-* |
|  | VEGF | | 133.10 [101.95–150.55] | 61.96 [39.14–99.60] | | 2.15 | 1.20 | 0.84 | *<0.001* |
|  | FGF-basic | | 61.64 [51.14-76.19] | 8.67 [6.70-12.13] | | 7.11 | 5.48 | 1.00 | *<0.001* |
|  | PDGF-bb | | 70.81 [56.08–107.12] | 25.94 [18.92–38.45] | | 2.73 | 1.66 | 0.89 | *<0.001* |

Cytokine-chemokine expression (pg/mL) is expressed in median [IQR] for patients with EV meningitis (restricted to PCR-positive results for EV in the cerebrospinal fluid (CSF EV+)) and the control group. Fold change, effect size, receiver operating characteristic (ROC) and p value between EV and control groups are indicated for each cytokine/chemokine.


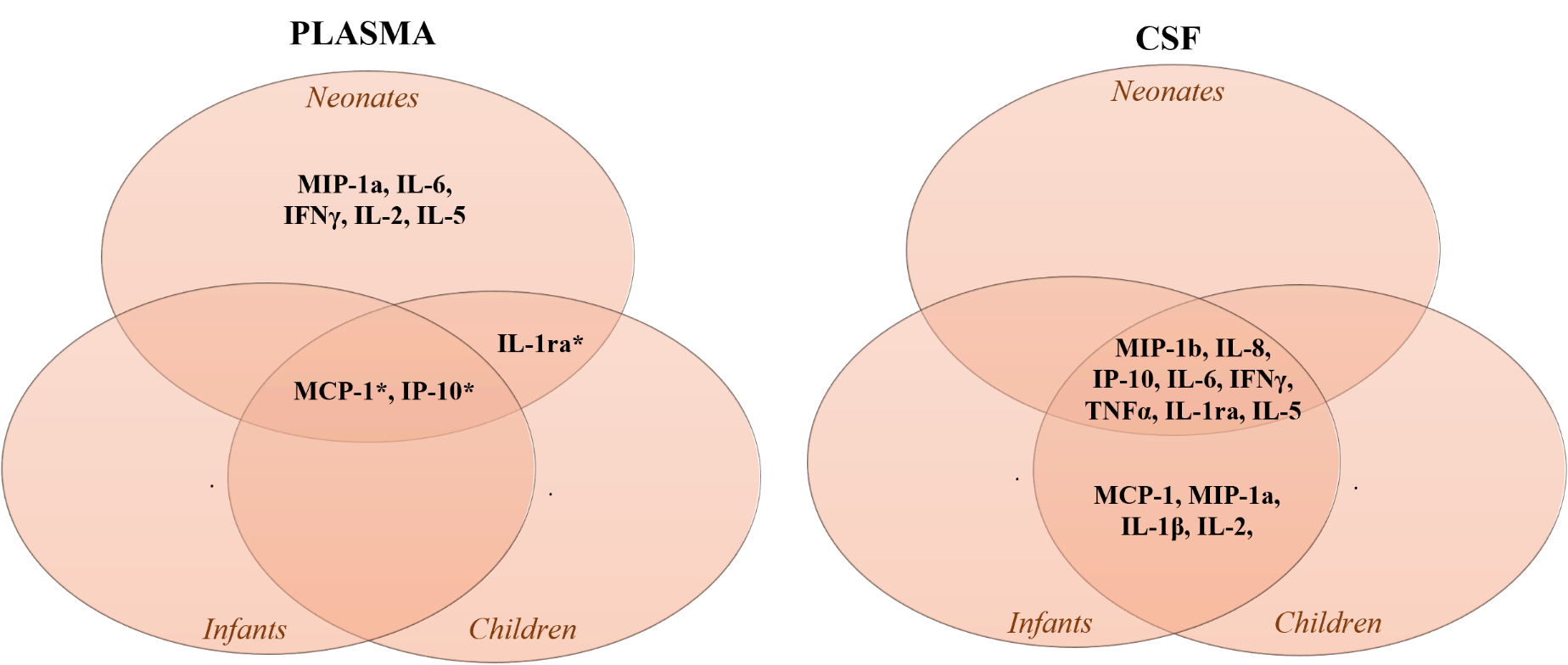


**Fig. S1.** Venn diagram showing the 12 selected cytokine/chemokines significantly over-expressed (or *down-expressed in children) shared between EV age groups (based on the fold change, effect size, ROC and p value analysis), in the plasma and the CSF (cerebrospinal fluid).

Table S5

Characteristics of EV neonates, infants and children with and without pleocytosis**.**

|  | NEONATES | | |  | INFANTS | | |  | CHILDREN | | |
| --- | --- | --- | --- | --- | --- | --- | --- | --- | --- | --- | --- |
|  | EV with pleocytosis  (n=6) | EV without pleocytosis  (n=10) | *p value* |  | EV with pleocytosis  (n=7) | EV without pleocytosis  (n=14) | *p value* |  | EV with  Pleocytosis  (n=50) | EV without pleocytosis  (n=5) | *p value* |
| Demographics |  |  |  |  |  |  |  |  |  |  |  |
| Age | 21 days [8d-27d] | 14 days [11d-23d] | *0.448* |  | 54 days [38d-82d] | 45 days [40d-85d] | *0.823* |  | 6 years [5y-8y] | 7 years [4y-8y] | *0.815* |
| Male sex | 3 [50%] | 6 [60%] | *0.549* |  | 6 (86%) | 8 (57%) | *0.210* |  | 39 (78%) | 4 (80%) | *0.702* |
| Sampling time |  |  |  |  |  |  |  |  |  |  |  |
| Time between onset of symptoms and lumbar puncture (hours) | 14 [2-23] | 5 [3-10] | *0.515* |  | 16 [9-51] | 15 [9-24] | *0.526* |  | 21 [15-57] | 14 [9-27] | *0.203* |
| Time between onset of symptoms and venepuncture (hours) | 19 [7-38] | 9 [6-18] | *0.233* |  | 14 [5-48] | 16 [9-26] | *0.794* |  | 25 [14-50] | 14 [8-25] | *0.178* |
| CSF profile |  |  |  |  |  |  |  |  |  |  |  |
| WBC (cell/mm^3^) | 795 [181-1210] | 2 [1-5] | *0.001* |  | 190 [68-510] | 1 [1-2] | < 0.001 |  | 110 [37-277] | 1 [1-2] | *<0.001* |
| Neutrophils and  Lymphocytes (%) | 36% - 64% | - | - |  | 41% - 59% | - | - |  | 50% - 50% | - | - |
| Proteins (g/L) | 0.9 [0.8-1.2] | 0.6 [0.5-0.6] | *0.002* |  | 0.7 [0.4-1.0] | 0.3 [0.2-0.4] | *0.004* |  | 0.3 [0.2-0.4] | 0.2 [0.2-0.2] | *0.003* |
| Glucose (mmol/L) | 2.1 [1.7-2.6] | 3.1 [2.7-3.2] | *0.007* |  | 2.7 [2.4-3.1] | 3.2 [2.8-3.5] | *0.021* |  | 3.6 [3.2-3.9] | 4.2 [3.9-4.8] | *0.037* |
| Blood profile |  |  |  |  |  |  |  |  |  |  |  |
| WBC (x 10^9^/L) | 11.3 [9.9-11.8] | 7.9 [5.7-9.1] | *0.020* |  | 11.2 [9.0-12.8] | 7.6 [5.4-11.7] | *0.101* |  | 10.5 [8.6-13.0] | 8.2 [6.5-11.6] | *0.270* |
| Neutrophils (x 10^9^/L) | 4.5 [2.2-7.3] | 4.3 [3.6-6.4] | *0.807* |  | 2.4 [1.9-4.5] | 2.8 [2.0-9.0] | *0.456* |  | 8.9 [7.1-11.5] | 7.1 [4.6-8.8] | *0.245* |
| Lymphocytes (x 10^9^/L) | 4.8 [4.5-4.9] | 1.9 [1.3-2.4] | *0.002* |  | 6.9 [5.3-9.1] | 2.6 [1.7-4.0] | *0.001* |  | 1.1 [0.8-1.5] | 1.4 [0.9-1.5] | *0.893* |
| Monocytes (x 10^9^/L) | 1.0 [0.9-1.6] | 0.6 [0.5-0.9] | *0.037* |  | 0.9 [0.6-1.2] | 0.6 [0.5-1.5] | *0.370* |  | 0.6 [0.5-0.8] | 0.7 [0.5-0.8] | *0.466* |
| C-reactive protein > 15 mg/L | 0 (0%) | 3 (30%) | *0.214* |  | 0 (0%) | 5 (36%) | *0.100* |  | 49 (98%) | 5 (100%) | *0.507* |
| EV characteristics |  |  |  |  |  |  |  |  |  |  |  |
| CSF viral load (log10 copy/ml) | 5.8 [4.2-6.4] | 4.6 [3.7-5.1] | *0.300* |  | 4.5 [3.3-4.9] | 4.7 [4.5-5.4] | 0.906 |  | 5.0 [4.3-5.7] | 4.9 [4.1-5.6] | *0.812* |
| Blood viral load (log10 copy/ml) | 3.8 [3.4-4.9] | 6.4 [5.5-8.0] | *0.009* |  | 5.1 [4.6-6.3] | 6.6 [4.3-7.2] | 0.459 |  | 3.8 [3.4-4.1] | 5.5 [3.3-6.0] | *0.266* |

Data are n (%) or median (IQR), unless otherwise indicated. Patients were included if the red blood cell count in the CSF was <160/mm3. EV: enterovirus; CSF: cerebrospinal fluid
